# Invariance of the Construct of Posttraumatic Stress Disorder: A Systematic Review

**DOI:** 10.1002/jts.22389

**Published:** 2019-04-03

**Authors:** Ateka A. Contractor, Stephanie V. Caldas, Megan Dolan, Prathiba Natesan, Nicole H. Weiss

**Affiliations:** 1 Department of Psychology, University of North Texas, Denton, Texas, USA; 2 Department of Educational Psychology, University of North Texas, Denton, Texas, USA; 3 Department of Psychology, University of Rhode Island, Kingston, Rhode Island, USA

## Abstract

We conducted a systematic review of studies that have evaluated invariance of the construct of posttraumatic stress disorder (PTSD) to summarize their conclusions related to invariance/noninvariance and sources of noninvariance. In November 2017, we searched Pubmed, PSYCINFO, PILOTS Web of Science, CINAHL, Medline, and Psychological and Behavioral Science Collection for abstracts and articles with these inclusionary criteria: peer-reviewed, including *DSM-IV* or *DSM-5* PTSD invariance as a main study aim, use of multigroup confirmatory factor analyses, and use of an independent PTSD instrument or module. In total, 45 articles out of 1,169 initially identified abstracts met inclusion criteria. Research assistants then followed Preferred Reporting Items for Systematic Reviews and Meta-Analyses (PRISMA) guidelines to complete a secondary search and independently extract data. Results indicated that *DSM-IV* dysphoric arousal and *DSM-5* hybrid model factors demonstrated the most stability; sources of instability were some intrusion (distress to trauma cues), dysphoria/numbing (traumatic amnesia, foreshortened future, emotional numbness, detachment), and arousal (hypervigilance) items. The PTSD Checklist and PTSD Reaction Index were most often used to assess PTSD in studies investigating its invariance; however, these measures demonstrated partial conceptual equivalence of PTSD across subgroups. Instead, clinician-administered measures demonstrated more conceptual equivalence across subgroups. Age, gender, cultural/linguistic factors, and sample diversity had the least moderating effect on PTSD’s symptom structure. Our review demonstrates the need to examine invariance of the PTSD construct following recommended guidelines for each empirical and clinical trial study to draw meaningful multigroup comparative conclusions.

Clinicians and researchers use diverse instruments to measure the psychological construct of posttraumatic stress disorder (PTSD). Scores obtained from these instruments are compared across subgroups, using statistical tests that depend on—yet assume—empirically established conceptual equivalence of the construct of PTSD. From a latent variable model perspective, participants with the same true PTSD factor (latent) score interpret and/or respond to PTSD-related items in a conceptually similar manner (i.e., same probability of observed scores), and PTSD items similarly capture the latent symptomology of PTSD across subgroups (Bauer, 2017; Dimitrov, 2010; Vandenberg & Lance, 2000; Wu, Li, & Zumbo, 2007). However, these assumptions may be violated. Interpretations of PTSD items across individuals matched on a PTSD latent score may differ due to subgroup attributes, developmental maturity, or intervention effects (Nolte, Elsworth, Sinclair, & Osborne, 2009; Putnick & Bornstein, 2016), wherein PTSD scores may not accurately reflect the latent symptomology of PTSD (Meredith, 1993). Hence, the conceptual equivalence of PTSD has to be examined versus assumed; we systematically reviewed studies that have investigated the conceptual equivalence of PTSD, with an aim to identify critical trends and future research avenues.

Invariance testing is increasingly used in psychological research (including that on PTSD) to investigate the conceptual equivalence and/or stability of a construct, the structural aspect of an instrument’s construct validity, and the clinical utility of results (Chen, 2008; Dimitrov, 2010). Measurement invariance is used to examine the associations between observed variables (i.e., responses to PTSD items) and the latent construct of PTSD in an effort to assess (a) comparability of the PTSD construct, (b) similarity in measuring the PTSD construct, and (c) whether PTSD items are defined identically across subgroups (Meredith, 1993). Measurement invariance permits a meaningful comparison of mean scores on measures of PTSD (e.g., severity scores) and of the relations between PTSD and other constructs across subgroups (Putnick & Bornstein, 2016). Structural invariance, which explores associations between latent variables, includes establishing invariance of factor variances–covariances and factor means (Byrne, Shavelson, & Muthén, 1989). Invariance testing targets parameters in an increasingly restrictive stepwise manner, as detailed later in this review (Byrne et al., 1989).

*Configural* or *form invariance* requires that the latent construct of PTSD is defined by identical PTSD item sets (i.e., a similar PTSD factor structure) across subgroups; the same number and/or type of PTSD factors are defined by the same number and/or type of PTSD items (e.g., Little & Slegers, 2005; Vandenberg & Lance, 2000). For example, across subgroups, PTSD is conceptualized by the same four symptom clusters according to the fifth edition of the *Diagnostic and Statistical Manual of Mental Disorders* (*DSM-5*; American Psychiatric Association [APA], 2013), and PTSD’s intrusion cluster is represented by the same five intrusion items. With configural noninvariance, for instance, the PTSD sleep item may load onto the intrusion factor in one subgroup only.

*Metric* or *weak factorial invariance* additionally requires consistent factor loadings for PTSD items across subgroups; each PTSD item is explained by the latent construct to a similar degree, and a 1 unit change in PTSD’s factor score is scaled to an equal unit change in the item score across subgroups (Gregorich, 2006; Putnick & Bornstein, 2016; Wu et al., 2007). With metric noninvariance, subgroups may differ in their understanding of PTSD items, or some PTSD items may be more applicable for one subgroup compared to another (Chen, 2008; Wu et al., 2007). For instance, metric noninvariance of the PTSD sleep symptom may indicate its stronger association with arousal in one subgroup only.

Next, *scalar/strong invariance* indicates that the response scale is used similarly and additionally requires equivalent item intercepts (i.e., the starting value of the PTSD scale on which the factor is built) across subgroups (Chen, 2008; Gregorich, 2006). Individuals matched on the PTSD factor score endorse similar response probabilities to PTSD-related items without bias or error (e.g., social desirability) differentially influencing these observed item scores (Dimitrov, 2010; Little & Slegers, 2005). For instance, scalar noninvariance of the PTSD hypervigilance item indicates that the mean hypervigilance score in one subgroup is unrelated to underlying PTSD severity (i.e., the factor score).

Further, *strict/error invariance* requires equivalent residual error variances and, thereby, equal precision and reliability in measuring PTSD items across subgroups (Dimitrov, 2010; Little & Slegers, 2005; Putnick & Bornstein, 2016). With strict noninvariance, PTSD items’ variance, or item uniqueness, is not consistently accounted for by common factors across subgroups (Chen, 2007). With strict invariance, investigators can meaningfully conduct structural invariance tests to compare between-group PTSD mean, variance, and covariance estimates (Bauer, 2017; Little & Slegers, 2005). Next, factor variances (i.e., the range of scores on PTSD’s latent factor) and covariances (i.e., intercorrelations between PTSD factor scores, or *phi invariance*) are examined for equivalence (Vandenberg & Lance, 2000); the information contained in each factor (variance) and the association between factors (covariance) need to be equal across subgroups. Finally, the equality of factor means is examined across subgroups; such invariance implies equality of population means on the latent PTSD variable or variables (Vandenberg & Lance, 2000). It is possible that two subgroups may have different latent means and covariances following measurement invariance; measurement invariance implies that the instrument is identically defined across subgroups such that structural invariance tests that compare factor-level statistics are valid.

It is critical to understand the function of invariance testing and interpretation of its results. First, there is a difference in the meaning and implications of subgroup differences (a) when examining the invariance of PTSD and (b) after establishing the invariance or noninvariance of PTSD. Not all subgroups will be invariant on the latent construct of PTSD; for example, trauma types and patterns may relate to complex PTSD in ways that are conceptually different from PTSD (Cloitre, Garvert, Weiss, Carlson, & Bryant, 2014) or to different PTSD symptom profiles (Contractor, Caldas, Fletcher, Shea, & Armour, 2018). When examining invariance, if subgroup differences influence the manner or type of PTSD item endorsement, the PTSD instrument no longer measures PTSD consistently and cannot be used to compare PTSD severity across subgroups based on bias (Meredith, 1993). After establishing (non)invariance, the presence of subgroup differences on PTSD scores informs to what they can be attributed and subsequent implications. With PTSD’s invariance, subgroup differences on PTSD scores may be attributed to true differences in PTSD’s latent construct. With respect to the noninvariance of PTSD, subgroup differences in PTSD scores may be attributed to sources other than differences in the latent construct of PTSD. For example, suppose the frequency with which detachment and sleep items are endorsed indicates PTSD severity in women whereas only the frequency with which the detachment item is endorsed indicates PTSD severity in men. The sleep item, then, is minimally associated with the latent construct of PTSD in men, which yields biased summed scores that cannot be meaningfully compared between men and women. In sum, subgroups may score differently on PTSD items (i.e., the probability of answering questions affirmatively); however, subgroups with similar levels of PTSD cannot have different probabilities of answering questions affirmatively that are conditional on their having similar PTSD latent scores—this is psychometrically impossible for an invariant PTSD instrument.

Second, invariance testing does not disregard PTSD symptom heterogeneity. Diagnosticians compute a summed PTSD score by giving equal importance to all items on a measure. However, within the factor-analytic framework used in invariance testing, PTSD items are weighted differently in the form of factor pattern coefficients, with PTSD’s latent construct influencing item responses. Thus, there is a difference in how a PTSD diagnosis is determined and how psychometric properties are examined when testing PTSD invariance. For instance, over time, an individual may consistently receive a PTSD diagnosis, such as scoring above an observed diagnostic cutoff on a PTSD instrument, and yet may endorse PTSD items differently (i.e., different symptom profiles), which can be attributed to differing maturity levels, item interpretations, or the latent construct of PTSD. In such situations, partial invariance can identify PTSD items that are influenced by factors beyond one’s inherent PTSD experience (Little & Slegers, 2005). Thus, although invariance testing allows for and acknowledges differences in PTSD symptom endorsements (i.e., observed PTSD item scores) across subgroups, if the aim is to measure the same PTSD construct for meaningful comparisons, the expected probability of PTSD item endorsements should be similar for a given PTSD level (i.e., latent factor) across subgroups.

Third, itis not always meaningful to examine or expect all or specific levels of invariance (Putnick & Bornstein, 2016); rather, the need for levels of invariance testing depends on study goals (Dimitrov, 2010). For instance, strict factorial invariance is not a prerequisite for meaningful comparisons of group means, or latent mean differences (Vandenberg & Lance, 2000), and only metric invariance is required for comparison of regression slopes (Chen, 2007). Given the multidimensional construct of PTSD, testing factorial covariance, versus factorial variance or factor mean invariance, may be of greater interest when the aim is to examine the stability of the dimensional structure of PTSD (Byrne & Shavelson, 1987). Relatedly, invariance testing draws from a latent variable model perspective; that is, an underlying PTSD latent variable causes PTSD symptom associations. Thus, invariance testing may not apply or may apply differently to diverse conceptual and statistical frameworks. For instance, network analysis conceptualizes the disorder of PTSD as a system of causally related and interacting symptoms (Borsboom & Cramer, 2013).

Finally, invariance and noninvariance results are informative (Putnick & Bornstein, 2016). Invariance results can speak to the conceptual equivalence of PTSD by referencing structural aspects of its construct validity (Dimitrov, 2010; Vandenberg & Lance, 2000) and can inform if moderating variables versus true PTSD construct differences are causing variations in how PTSD symptoms are clustered or expressed (Rasmussen, Verkuilen, Ho, & Fan, 2015; Yuan & Chan, 2016). Also, invariance results can inform psychometric evaluations of PTSD instruments. By assessing how subgroups differ in their responses to PTSD-related items, problematic and noninvariant items on an instrument that measures PTSD symptoms can be identified to inform the instrument’s modification; such steps can reduce bias and improve the instrument’s accuracy (Kim, Yoon, & Lee, 2012; Sass, 2011). Statistically, the use of a noninvariant instrument to assess PTSD, especially without accounting for noninvariance sources, could bias diagnostic screening and validity, PTSD diagnosis and corresponding prevalence rates, PTSD symptom score means, and other estimates derived from PTSD item scores, and, thus, associations of PTSD scores and/or estimates with other variables cannot be meaningfully compared across subgroups (Hall, Elhai, Grubaugh, Tuerk, & Magruder, 2012). Lastly, invariance results can have implications in terms of policy and resource-allocation (Meredith & Teresi, 2006); resource allocation for implementing PTSD interventions is based on comparative findings of PTSD severity in the treatment versus control conditions. Thus, our systematic review identified studies that assessed conceptual equivalence of the construct of PTSD and summarized findings regarding PTSD invariance or noninvariance as well as the source or sources of any identified noninvariance.

## Method

Following Preferred Reporting Items for Systematic Reviews and Meta-Analyses (PRISMA) guidelines (Moher, Liberati, Tetzlaff, Altman, & Prisma Group, 2009), we searched the following databases on November 11, 2017: Pubmed, PSYCINFO, Published International Literature on Post Traumatic Stress, Web of Science, Cumulative Index of Nursing and Allied Health Literature, Medline, and Psychological and Behavioral Science Collection. Search terms included a combination of *PTSD*, *posttraumatic stress disorder*, *measurement invariance*, *factorial invariance*, *structural invariance*, *factorial stability*, *factor structure*, *latent structure*, *symptom structure*, *crossvalidation*, *structure model*, *comparison*, *confirmatory factor analysis, multiple-group*, *multi-sample*, *invariance*, and *stability*. Inclusion criteria were: (a) English-language publication in a peer-reviewed journal, (b) examination of PTSD invariance (based on *DSM-IV* or *DSM-5*) as a primary study aim, (c) measurement of PTSD using a standalone instrument or a diagnostic interview module, and (d) evaluation of invariance in a multigroup confirmatory factor analytical (MGCFA) approach (Byrne et al., 1989; Millsap & Everson, 1993). We used an MGCFA approach (vs. a multiple–indicators multiple–causes [MIMIC] model) because it examines the equivalence of all aspects of the PTSD symptom structure (e.g., factor variances and covariances) simultaneously (Little & Slegers, 2005). The MIMIC model requires rather than determines configural invariance and assumes rather than tests equal factor loadings and homogeneity of residual variances across subgroups (Bauer, 2017; Kim et al., 2012). Two independent teams, each with two trained research assistants, screened abstracts that were identified during the initial search, and two coauthors examined the remaining articles for inclusionary criteria. A secondary reference search was completed on the final list of articles. Two research assistants independently used standardized coding forms to extract data.

## Results

**Figure 1.**
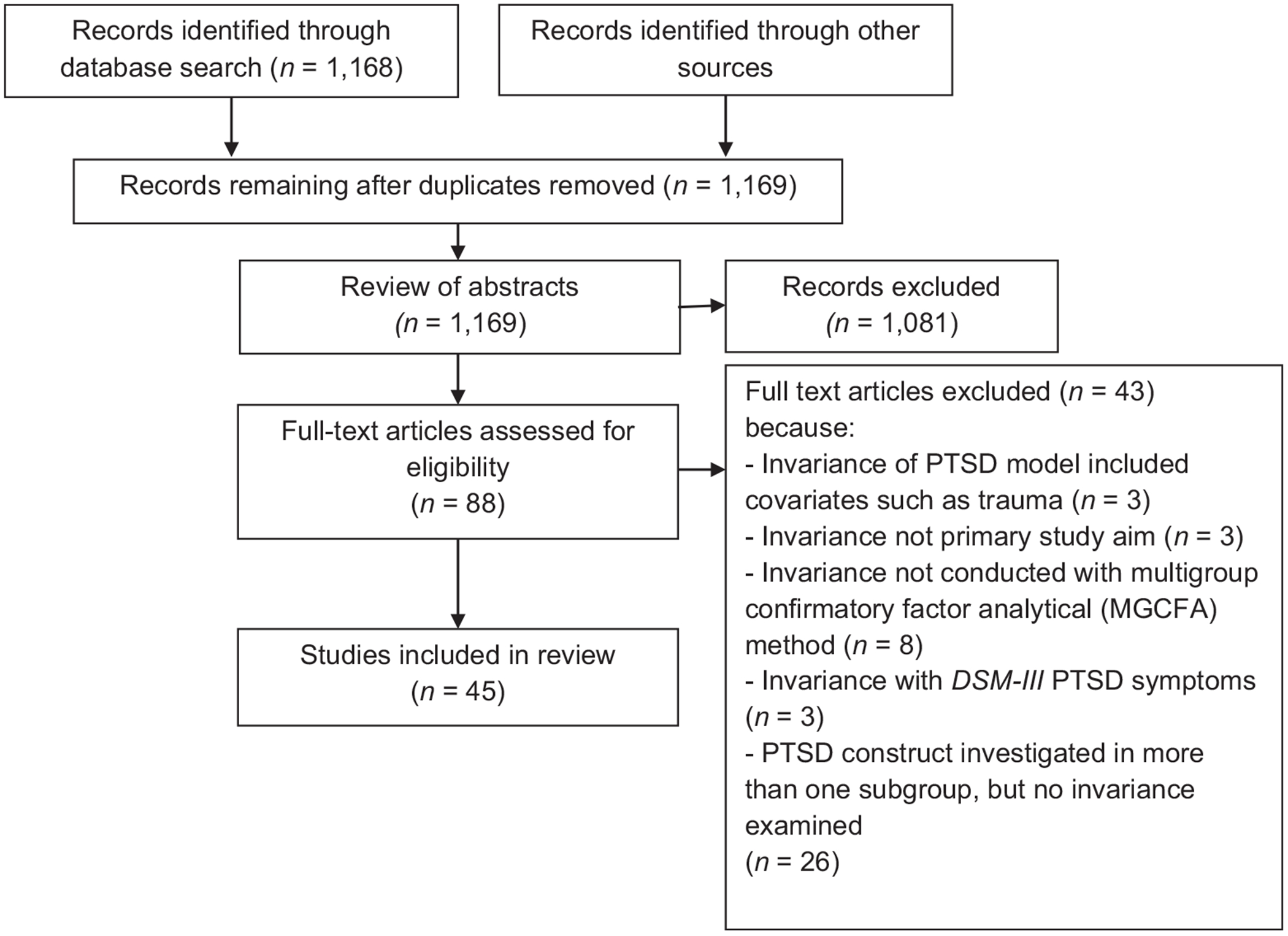
Outlined procedure for the systematic review. PTSD = posttraumatic stress disorder.

After removing duplicates, the initial search resulted in 1,168 unique articles; one additional article was identified through secondary screening (Elhai, Biehn, Naifeh, & Frueh, 2011), for a total of 1,169 unique articles. Two research assistants reviewed abstracts and excluded 1,081 articles. Next, 88 full-text articles were reviewed by two coauthors, and 45 articles were identified as meeting inclusionary criteria (Figure 1). For this review, each multigroup (MG) analysis indicated one comparative invariance analysis of the PTSD construct across subgroups (e.g., male vs. female participants). A total of 96 MG analyses were included in this review.

### Sample Descriptors, PTSD Measurement, and Subgroups

Supplemental Table S1 outlines sample descriptors. There were 61 MG analyses (63.5%) that utilized United States-based samples in this group, 35 MG analyses (36.5%) included predominantly (> 60%) White participants. Six MG analyses (6.7%) examined all-male samples, and five MG analyses (5.6%) examined all-female samples. The majority ofMG analyses (*n* = 72, 75.0%) utilized adult samples. Most MG analyses utilized *DSM-IV* criteria (*n* = 83, 86.5%), self-report measures (*n* = 86, 89.6%), and the PTSD Checklist (PCL; Weathers, Litz, Herman, Huska, & Keane, 1993; Weathers et al., 2013; *n* = 51, 53.1%). Most MG analyses (*n* = 81, 84.4%) examined differences across two subgroups, and most MG analyses examined subgroups based on time points that followed traumatic events (TE; *n* = 17, 17.7%), gender (*n* = 16, 16.7%), and types or exposure levels of TE (*n* = 14, 14.6%). For the latter, Hetzel-Riggin (2009) examined different trauma types; Zelazny and Simms (2015) examined Criterion A versus non-Criterion A TE; Frankfurt, Armour, Contractor, and Elhai (2016) examined direct versus indirect trauma exposure; Saul, Grant, and Carter (2008) examined violent versus nonviolent stressors; and Elhai et al. (2009) examined reference to a worst TE versus no reference to a worst TE versus reference to overall trauma history. Fewer MG analyses examined subgroups based on cultural and/or linguistic status (*n* = 13, 13.5%), deployment-related time points (*n* = 8, 8.3%), diverse samples (*n* = 8, 8.3%), deployment status (*n* = 6, 6.3%), psychopathology- and diagnosis-related variables (*n* = 5, 5.2%), administration mode and types of measures (*n* = 4, 4.2%), age (*n* = 3, 3.1%), educational attainment (*n* = 1, 1.0%), and intervention time points (*n* = 1, 1.0%).

### Methodological Variations in Invariance Testing

#### PTSD factor-analytical models.

Supplemental Tables S2 and S3 outline item mappings of the *DSM-IV* and *DSM-5* PTSD factor-analytical models, respectively, and Supplemental Table S4 outlines statistical aspects related to the invariance testing. Referencing *DSM-IV* criteria, the three-factor model had little empirical support (Elhai & Palmieri, 2011). A four-factor emotional numbing (EN) model (King, Leskin, King, & Weathers, 1998), similar to a model presented by Sack, Seeley, and Clarke (1997), separated PTSD-related avoidance and numbing into two factors. A four-factor dysphoria model created a nonspecific dysphoria factor (Simms, Watson, & Doebbeling, 2002). Finally, a five-factor dysphoric arousal (DA) model separated the arousal cluster into dysphoric and anxious arousal symptoms (Elhai, Biehn, Armour et al., 2011). Referencing *DSM-5* criteria, the four-factor *DSM-5* model, which includes intrusions, avoidance, negative alterations in cognitions and mood (NACM), and alterations in arousal and reactivity (AAR), closely resembles the *DSM-IV* EN model (Friedman, Resick, Bryant, & Brewin, 2011). The *DSM-5* dysphoria (Miller et al., 2013) and DA (Elhai, Biehn, Armour et al., 2011) models are similar to their *DSM-IV* counterparts. A six-factor anhedonia (AN) model differentiated NACM symptoms of negative from positive affect (Liu et al., 2014) whereas a six-factor externalizing behavior (EB) model proposed an EB factor representing emotional dysregulation (Tsai et al., 2015). A sevenfactor hybrid model integrated other *DSM-5* model components (Armour et al., 2015). Although the invariance literature includes modifications to these PTSD models, it was not feasible or meaningful to account for every model modification, and we report herein the most widely researched factor categorizations. Six MG analyses (6.3%) examined invariance of the one-factor PTSD model whereas 90 MG analyses (93.8%) examined the invariance of PTSD clusters. Regarding *DSM-IV*, most MG analyses examined the EN (*n* = 33, 34.4%) or dysphoria (*n* = 25, 26.0%) models, 11 MG analyses (11.5%) examined the DA model, and six MG analyses (6.3%) examined the three-factor model. In terms of the *DSM-5*, five MG analyses (5.2%) examined the *DSM-5* model, two MG analyses (2.1%) examined the EB and AN models, and five MG analyses (5.2%) examined the hybrid model. The recent release of *DSM-5* diagnostic criteria and corresponding introduction of *DSM-5* models accounted for the few MGs that investigated *DSM-5* (*n* = 13, 13.5%) versus *DSM-IV* (*n* = 83, 86.5%) models. Most MG analyses (*n* = 75, 78.1%) compared the fit of PTSD models and subsequently subjected the optimal model to invariance testing.

#### Invariance model(s).

In Model A, subgroups vary on all parameter estimates (configural/form). Models B, C, and D further constrain factor loadings (metric/pattern/weak/factorial), item intercepts (scalar/strong factorial), and residual error variances (strict factorial), respectively, across subgroups. Model D constraints do not apply to subsequently tested models. Model E, which constrains factor variances and covariances (ϕ) is tested against Model C, whereas Model F, which constrains factor means, is tested against Model E (Gregorich, 2006; Meredith & Teresi, 2006). With noninvariance, partial invariance, which is assessed by freeing constraints that caused poor model fit, is established before examining further levels of invariance (Little & Slegers, 2005; Vandenberg & Lance, 2000).

In total, 24 (25.0%) MG analyses examined all invariance levels, and 33 (34.4%) examined three invariance levels. For noninvariance, only 17 MG analyses (17.7%) examined partial invariance. For the one-factor PTSD model, two MG analyses revealed one intrusion item (acting/feeling as if TE was recurring) and two arousal items (sleep and concentration difficulties) as scalar noninvariance sources, whereas two MG analyses indicated avoidance and numbing parcel factor loadings and intercept values as scalar noninvariance sources. For the *DSM-IV* model factors, metric noninvariance sources were intrusion items, whereas the factor covariance noninvariance source was the intrusion–avoidance cluster covariance (one MG analysis each). For the *DSM-IV* dysphoria model factors, metric noninvariance sources included intrusion items assessing intrusive memories and distress upon encountering TE cues; dysphoria/numbing items assessing detachment, diminished interest, traumatic amnesia, restricted affect, foreshortened future, and anger; and the arousal item assessing hypervigilance. For the *DSM-IV* dysphoria model factors, items assessing detachment, diminished interest, and traumatic amnesia also contributed to scalar nonvariance. For the *DSM-IV* EN model factors, metric noninvariance sources were intrusion items that assess physiological distress with TE cues; numbing items that assess traumatic amnesia, foreshortened future, emotional numbness, and reduced positive affect; avoidance items; and arousal items that assess startle and hypervigilance. Items that assess foreshortened future, startle, and psychological distress with TE cues contributed to scalar noninvariance. Arousal–avoidance factor covariance contributed to factor covariance noninvariance.

#### Invariance model comparison indices.

Generally, each invariance model is first described with individual fit indices. A significant chi-square value indicates a good model fit. The comparative fit index (CFI), Tucker–Lewis Index (TLI), root mean square error of approximation (RMSEA), and standardized root mean square residual (SRMR) values are used to counter the tendency for chi-square to be significant with larger samples (Hu & Bentler, 1999). To compare invariance models, chi-square change (Δχ^2^), ΔCFI, ΔRMSEA, and/or ΔSRMR values are used (Cheung & Rensvold, 2002; Gregorich, 2006). In our review, 83 MG analyses (86.5%) reported the Δχ^2^ criterion; 30 MG analyses (31.3%) additionally reported ΔCFI criterion. There were 12 MG analyses (12.5%) that did not report change values. Only five MG analyses (5.2%) reported conceptualization of PTSD symptoms as continuous indicators, and all of these analyses used maximum likelihood (ML)-based estimators as recommended (Rhemtulla, Brosseau-Liard, & Savalei, 2012). Authors of most MG analyses reported using ML-based estimators (*n* = 71, 74.0%) as opposed to mean- and variance–adjusted weighted least squares (WLSMV; *n* = 15, 16.6%). More WLSMV versus ML-based MG analyses compared baseline models to determine the optimal model in each subgroup (93.3% of WLSMV vs. 73.2% of ML-based MGs); conducted missing data analyses (73.3% of WLSMV vs. 43.7% of ML-based MGs); examined partial invariance (26.7% of WLSMV vs. 15.5% of ML-based MGs); and found scalar (85.7% of WLSMV vs. 67.6% of ML-based MGs), strict (75% of WLSMV vs. 39.4% of ML-based MGs), and factor variance/covariance invariance (66.7% of WLSMV vs. 47.4% of ML-based MGs). Three of the five MG analyses (5.2%) that used binary variables tested metric invariance separately.

### Invariance Testing Summary

#### PTSD factor-analytical models.

Supplemental Table S5 provides percentages that reflect the count of MG analyses that demonstrated a particular invariance level compared to the count of MG analyses examining a particular invariance level. The *DSM-IV* three-factor, EN, and dysphoria model parameters were examined for the most invariance levels. For *DSM-IV*, the DA model demonstrated invariance at most levels: 11 (100.0%), 10 (90.9%), seven (70.0%), eight (80.0%), eight (100.0%), and four (57.1%) MG analyses found invariance of Models A–F, respectively. In terms of *DSM-5*-based models, the hybrid model demonstrated the most invariance (excluding Model F invariance), the AN model factors demonstrated invariance with Models A–C, and two MG analyses (100.0%) failed to support even the minimum level of invariance (Model A) for the EB model factors.

#### PTSD measures.

Among self-report measures, the PCL (Weathers et al., 1993, 2013) and UCLA Posttraumatic Stress Disorder Reaction Index (PTSD-RI; Steinberg, Brymer, Decker, & Pynoos, 2004) were examined for the largest number of invariance levels. For the PCL, 47 (92.2%), 36 (76.6%), 12 (34.3%), 10 (41.7%), 12 (42.9%), and two (14.3%) MG analyses found invariance with Models A–F, respectively. Partial invariance for Models B, C, and E was also supported. For the PTSD-RI, eight (100.0%), four (50.0%), three (50.0%), three (50.0%), four (57.1%), and four (66.7%) MG analyses found invariance with Models A–F, respectively. Although only one MG analysis each examined invariance of the Posttraumatic Stress Disorder Questionnaire (PTSD-Q; Cross & McCanne, 2001) and the Psychological Reactions following International Missions (PRIM; Andersen, 1998) measures, both demonstrated invariance at all tested levels (Models A, B, D, and E invariance for the PTSD-Q, and Models A–C invariance for the PRIM).

Regarding clinician-administered measures, the Clinician Administered PTSD Scale (CAPS; Weathers, Keane, & Davidson, 2001) demonstrated invariance for Models A (*n* = 2, 66.7%), B (*n* = 3, 100.0%), and Partial C (*n* = 2, 100.0%). For the Diagnostic Interview Schedule–PTSD module (Kilpatrick & Saunders, 1997), only Model A invariance (*n* = 3; 100.0%) was supported (Models A and B were examined). For the National Survey of Adolescents–Replication PTSD module (Resnick, Kilpatrick, Dansky, Saunders, & Best, 1993), two (100.0%) MG analyses found invariance for Models A–E and noninvariance for Model F. For the Mini-International Neuropsychiatric Interview 6 (M.I.N.I.–6) PTSD module (Sheehan et al., 1998; Zelazny & Simms, 2015), two (100.0%) MG analyses found Models A–C invariance.

#### Subgroup comparisons.

Most subgroups were examined for all or most invariance levels except in terms of variables that dealt with deployment time point, education, and intervention time point. Age, gender, cultural and/or linguistic status, and diversity of samples demonstrated invariance at all or most assessed invariance levels, and more than half of the MG analyses found invariance at all levels or excluding one level. For age, three (100.0%), two (66.7%), one (50.0%), one (50.0%), two (100.0%), and one (100.0%) MG analyses found invariance for Models A–F, respectively. For gender, 14 (87.5%), 11 (78.6%), seven (58.3%), six (85.7%), eight (88.9%), and one (11.1%) MG analyses found invariance for Models A–F, respectively. For cultural and/or linguistic comparisons, 12 (92.3%), seven (77.8%), three (33.3%), two (100.0%), three (50%), zero (0%), two (66.7%), one (100.0%), and two (100.0%) MG analyses found invariance for Models A, B, C, Partial C, D, Partial D, E, Partial E, and F, respectively. Finally, for diverse samples, eight (100.0%), five (62.5%), one (100.0%), one (100.0%), two (66.7%), and one (100.0%) MG analyses found invariance for Models A, B, Partial B, C, E, and Partial E, respectively.

## Discussion

### Invariance Results: One-Factor PTSD Model

Our findings highlight discrepancies between the scientific value and clinical applicability of invariance research. Few reviewed studies examined invariance of the overall PTSD construct (one-factor PTSD model); however, several existing empirical studies and clinical trials compared total PTSD scores corresponding to the overall PTSD construct, rather than PTSD subscale scores corresponding to multiple-factor PTSD models (e.g., Foa et al., 2018). Further, the limited research we reviewed indicated that caution should be observed when comparing the overall PTSD construct across certain subgroups. Although all analyses found configural invariance (i.e., consistency in the pattern and strength of factor loadings on an overarching PTSD factor across subgroups), some analyses indicated metric noninvariance; that is, there may have been different observed scores despite identical underlying levels of PTSD severity (Cheung & Rensvold, 2002; Meredith & Teresi, 2006). Overall, additional research that examines invariance of the widely used one-factor PTSD model is needed to develop definitive conclusions regarding its clinical utility.

### Invariance Results: Multiple-Factor PTSD Models

The *DSM-IV* DA and *DSM-5* hybrid model factors demonstrated invariance at most levels compared to their respective counterparts and clinically used models (i.e., the *DSM-IV* three-factor and *DSM-5* four-factor models). Consistent with existing empirical support (Armour, Mullerova, & Elhai, 2016; Contractor, Greene, Dolan, & Elhai, 2018), both models demonstrated a stable and comparable representation of the PTSD symptom structure (i.e., the structural aspect of construct validity). In addition, our findings support further investigation of the recently proposed and debated hybrid model and its clinical utility (Silverstein, Dieujuste, Kramer, Lee, & Weathers, 2017). Notably, our conclusions are limited by the number of MG analyses that examined a PTSD model, particularly for *DSM-5*, and levels of invariance tested. For instance, although the AN model factors demonstrated invariance for Models A–C indicating a promising potential for conceptual equivalence, no further levels of invariance were tested. Additionally, it is unclear if the number of indicators that define a factor could have influenced invariance results. Several *DSM-IV* DA (avoidance, anxious arousal) and *DSM-5* hybrid (avoidance, externalizing behaviors, anxious arousal, dysphoric arousal) model factors include only two items, which could reduce factor stability despite enhancing domain homogeneity (Palmieri, Weathers, Difede, & King, 2007) because it is easier to find significant correlations between two variables even if they do not reflect an underlying factor. Thus, two substantively unrelated but statistically correlated items may or may not be indicators of a common construct; such a situation may negatively impact invariance. Additionally, for invariance testing, investigators need to ascertain that the referent indicator for a latent factor does not result in differential item functioning (Dimitrov, 2010). This issue is more salient when two items define a latent construct. Thus, correspondence between the number of indicators that define a factor and invariance needs empirical investigation.

For the most part, studies that examined the invariance of *DSM-IV* as compared to *DSM-5* models demonstrated a higher level of statistical rigor. More *DSM-IV* (versus *DSM-5*) MG analyses compared baseline models to determine the optimal and parsimonious model in each subgroup separately (59.0% of *DSM-IV* vs. 30.8% of *DSM-5*), reported a specific method to account for missing data (e.g., 31.3% of *DSM-IV* vs. 23.1% of *DSM-5* MGs reported using ML-based estimators), and examined partial invariance (22.9% of *DSM-IV* vs. 0% of *DSM-5*). However, more *DSM-5* (76.9%) MG analyses used the recommended ΔCFI and Δχ^2^ values to determine invariance compared to *DSM-IV* (51.8%) MG analyses. Notably, 6.0% of *DSM-IV* compared with 0% of *DSM-5* MG analyses reportedly treated PTSD variables as continuous, using ML-based estimators as recommended. There is mixed support for treating a five-option item as categorical or continuous contingent on sample and model parameter characteristics (Babakus, Ferguson, & Jöreskog, 1987; Rhemtulla, Brosseau-Liard, & Savalei, 2012). Relatedly, MG analyses that used WLSMV demonstrated a higher level of statistical rigor and higher levels of invariance compared to MG analyses that used ML-based estimators. Notably, only one study, which was based on *DSM-IV* symptoms, with binary variables examined metric invariance against recommendations (Putnick & Bornstein, 2016). Future *DSM-5* invariance studies should address these issues of statistical rigor, particularly given the growing role invariance testing is likely to play in shaping future *DSM* revisions.

### Invariance Results: Partial Invariance

Of statistical concern, most studies continued to examine further invariance upon finding noninvariance at an earlier stage (i.e., metric invariance). Invariance testing is a hierarchical process, and each level of invariance depends on the prior level. The best practice in the case of noninvariance is to examine partial invariance to inform the next steps contingent on the research and/or clinical rationale for invariance testing, practicality, and intended use of the measure (Millsap & Kwok, 2004). Removal of noninvariant items from the PTSD assessment instrument, although possible, may reduce PTSD domain coverage, heterogeneity, and the clinical relevance or utility of being able to endorse different PTSD items to qualify for a PTSD diagnosis; such consequences render this to be the least practical alternative (Bauer, 2017). Alternatively, investigators can account for noninvariance in statistical computations to maximize the accuracy and meaning of conclusions (Bauer, 2017), modify the PTSD assessment instrument, or abandon using that particular PTSD assessment instrument for the subgroups under consideration (Millsap & Kwok, 2004). At the very minimum, one could aim for configural invariance and appropriately deal with any further obtained noninvariance in PTSD research.

Our review indicated trends in the type of PTSD items that contribute to construct instability contingent on—and limited by—the examined nature of subgroups, PTSD model, and invariance levels. Overall, intrusion cluster symptoms involving intrusive memories and distress to TE cues; dysphoria/numbing cluster symptoms of traumatic amnesia, anhedonia, foreshortened future, emotional numbness/restricted affect, and detachment; and the arousal-cluster symptom of hypervigilance were unstable. Most items (anhedonia, distress to TE cues, intrusive memories, traumatic amnesia, foreshortened future, emotional numbness, detachment, and hypervigilance) differed in loadings onto respective PTSD factors across subgroups differentiated by deployment variables (e.g., pre vs. postdeployment). Occurrence of deployment and passage of time thereafter may influence an individual’s interpretation of these PTSD items, possibly due to the psychological impact of deployment, including postdeployment changes in worldview (Hoge, Auchterlonie, & Milliken, 2006; Newby et al., 2005), and these non-invariant PTSD items should be accounted for in research that compares subgroups differentiated on deployment variables.

Our review found that responses to items that assess foreshortened future and traumatic amnesia were likely to be influenced by characteristics other than underlying PTSD severity, such as comorbid pain, administration mode of the PTSD assessment instrument, or time since deployment. Such findings concur with existing literature that has questioned their significance within PTSD symptomatology (Armour, Contractor, Shea, Elhai, & Pietrzak, 2016; Friedman et al., 2011), which possibly contributed to modification of the foreshortened future item in the *DSM-5* (APA, 2013). Notably, our results showing that most numbing/dysphoria (e.g., anhedonia) items contributed to PTSD construct nonequivalence add to the existing debate on their specificity to the PTSD construct (Contractor, Greene et al., 2018). Removing or modifying these items may increase the validity of PTSD assessment instruments for certain subgroups (Sass, 2011).

### Invariance Results: PTSD Instruments and Nature of Subgroups

Although the PCL and PTSD-RI were the most widely researched instruments at several invariance levels, they demonstrated mainly configural and metric invariance. Thus, even widely used PTSD measures should first be examined for invariance prior to comparing PTSD estimates derived from item scores across subgroups. Despite limited data, measures such as the PRIM and PTSD-Q showed promise in measuring a stable PTSD construct; that is, they demonstrated invariance at all assessed levels. Additional invariance research needs to be conducted on these measures. Albeit with limited data, clinician-administered PTSD measures and self-report PTSD measures, excluding self-report measures administered as interviews, have demonstrated equivalent configural and metric invariance of the assessed PTSD construct. However, clinician-administered PTSD measures have demonstrated stricter invariance levels (e.g., scalar, strict factorial, factor variance/covariances excluding factor means) compared to self-report PTSD measures. Despite these results, sound psychometric properties, and the ability to minimize misinterpretations (Foa & Tolin, 2000; Weathers et al., 2017), it is concerning that clinician-administered PTSD measures, such as the CAPS, and diagnostic interview PTSD modules, such as the M.I.N.I., have been subjected to less invariance testing.

Regarding subgroup comparisons, for the most part, configural invariance was supported, indicating, that the PTSD factor structure, despite PTSD model diversity, is quite stable and comparable. Our results primarily indicated less moderating effects of variables such as age (examined in one study), gender, cultural or linguistic factors, and sample diversity. In contrast, individuals in subgroups distinguished by administration mode or type of measures, psychopathology and diagnostic-related variables, deployment status (absence vs. presence), and educational attainment status may not be responding to PTSD items based on true differences in the PTSD latent construct. Surprisingly, only one study examined PTSD invariance in a sample that was receiving intervention; such studies are a precursor to valid interpretations of clinical trial data (Baschnagel, O’Connor, Colder, & Hawk, 2005).

Some limitations should be considered in terms of our findings. First, methodological variations in invariance testing across studies limited our ability to draw definite conclusions. Second, studies that used alternate invariance testing approaches based on item-response theory (Millsap & Everson, 1993) or MIMIC model approaches (Joreskog & Goldberger, 1975) were excluded to maintain a meaningful scope of our review; this can be addressed in future research. Notably, we observed some similar findings among reviewed MGCFA studies and nonreviewed MIMIC model studies. For instance, when comparing the PCL across English and Spanish languages, Marshall (2004) used MGCFA and found invariance of Models A–C and E. Similarly Miles, Marshall, and Schell (2008) used a MIMIC model and found almost no differential item functioning. Further, Hall and colleagues (2012) used a MIMIC model to compare the PCL across genders and found noninvariance of Models C–E compared to the majority of our reviewed studies, which similarly indicated Model C noninvariance and differently indicated Model D–E invariance. Relatedly, future research could investigate nonpublished papers to address filedrawer effects. Third, there is a need to examine invariance across diverse subgroups, such as those characterized by different trauma types—a variable shown to significantly impact posttrauma outcomes (Contractor, Caldas et al., 2018).

Fourth, invariance testing has limited generalizability in that the applicability of results is limited by the foundational latent variable model perspective as well as the nature of the PTSD instrument, of the subgroups, and of the PTSD models examined in the reviewed studies. Thus, we need several replication studies to draw generalizable conclusions. Conducting a meta-analysis would help derive numerical estimates of between-model significance and corresponding important implications. Fifth, the smaller number of studies that examined *DSM-5* symptoms essentially limited the scope of our review and provided only preliminary findings for *DSM-5* PTSD invariance. Finally, we could not address debated issues in the invariance literature, such as statistical thresholds for detecting practical construct differences and invariance violations (Putnick & Bornstein, 2016). Examining invariance and accounting for noninvariance can further scientific inquiry of the measurement and structural aspects of the construct validity of PTSD assessment instruments as well as of subgroup differences in PTSD symptom expression. This is necessary to establish that the components and the item-to-factor relations are identical across subgroups before conducting latent-level comparisons. Further investigations of PTSD invariance is to be an ongoing effort for the trauma research and clinical community.

## Supplementary Material

Supplemental Material

## References

[R1] American Psychiatric Association. (2013). Diagnostic and statistical manual of mental disorders (5th ed.). Washington, DC: Author.

[R2] Andersen HE (1998). Danske FN-soldater 2 år efter: Opfølgn ingsundersøgelse af DANBAT hold 7 og 8 [Danish UN solders 2 years after: A follow-up investigation of rotation 7 and 8 of the DANBAT force]. Copenhagen: Foryarets Center for Leadership (FCL) Publication no. 144.

[R3] Armour C, Contractor AA, Shea MT, Elhai JD, & Pietrzak RH (2016). Factor structure of the PCL-5: Relationships among symptom clusters, anger, and impulsivity. Journal of Nervous and Mental Disease, 204, 10–115. 10.1097/NMD.0000000000000430 26669984

[R4] *Armour C, Elhai JD, Layne CM, Shevlin M, Duraković-Belko E, Djapo N, & Pynoos RS (2011). Gender differences in the factor structure of posttraumatic stress disorder symptoms in war-exposed adolescents. Journal of Anxiety Disorders, 25, 604–611. 10.1016/j.janxdis.2011.01.010 21377317

[R5] *Armour C, Layne CM, Naifeh JA, Shevlin M, Duraković-Belko E, Djapo N, … Elhai JD (2011). Assessing the factor structure of posttraumatic stress disorder symptoms in war-exposed youths with and without Criterion A2 endorsement. Journal of Anxiety Disorders, 25, 80–87. 10.1016/j.janxdis.2010.08.006 20822881

[R6] Armour C, Mullerova J, & Elhai JD (2016). A systematic literature review of PTSD’s latent structure in *the Diagnostic and Statistical Manual of Mental Disorders: DSM-IV to DSM-5*. Clinical Psychology Review, 44, 60–74. 10.1016/j.cpr.2015.12.003 26761151

[R7] Armour C, Tsai J, Durham TA, Charak R, Biehn TL, Elhai JD, & Pietrzak RH (2015). Dimensional structure of *DSM-5* posttraumatic stress symptoms: Support for a hybrid Anhedonia and Externalizing behaviors model. Journal of Psychiatric Research, 61, 106–113. 10.1016/j.jpsychires.2014.10.012 25479765

[R8] *Asmundson GJ, Wright KD, McCreary DR, & Pedlar D (2003). Post-traumatic stress disorder symptoms in United Nations peacekeepers: An examination of factor structure in peacekeepers with and without chronic pain. Cognitive Behaviour Therapy, 32, 26–37. 10.1080/16506070310003648 16291532

[R9] Babakus E, Ferguson CE Jr., & Jöreskog KG (1987). The sensitivity of confirmatory maximum likelihood factor analysis to violations of measurement scale and distributional assumptions. Journal of Marketing Research, 222–228. 10.2307/3151512

[R10] *Baschnagel JS, O’Connor RM, Colder CR, & Hawk LW (2005). Factor structure of posttraumatic stress among western New York undergradutes following the September 11th terrorist attack on the World Trade Center. Journal of Traumatic Stress, 18, 677–684. 10.1002/jts.20076 16382430

[R11] Bauer DJ (2017). A more general model for testing measurement invariance and differential item functioning. Psychological methods, 22, 507–526. 10.1037/met0000077 27266798 PMC5140785

[R12] *Bennett DC, Kerig PK, Chaplo SD, McGee AB, & Baucom BR (2014). Validation of the five-factor model of PTSD symptom structure among delinquent youth. Psychological Trauma Theory Research Practice and Policy, 6, 438–447. 10.1037/a0035303

[R13] *Biehn TL, Elhai JD, Fine TH, Seligman LD, & Richardson JD (2012). PTSD factor structure differences between veterans with and without a PTSD diagnosis. Journal of Anxiety Disorders, 26, 480–485. 10.1016/j.janxdis.2012.01.008 22387183

[R14] *Boal AL, Vaughan CA, Sims CS, & Miles JN (2017). Measurement invariance across administration mode: Examining the Posttraumatic Stress Disorder (PTSD) Checklist. Psychological Assessment, 29, 76–86. 10.1037/pas0000301 27054619

[R15] Borsboom D, & Cramer AO (2013). Network analysis: An integrative approach to the structure of psychopathology. Annual Review of Clinical Psychology, 9, 91–121. 10.1146/annurev-clinpsy-050212-185608 23537483

[R16] Byrne BM, & Shavelson RJ (1987). Adolescent self concept: Testing the assumption of equivalent structure across gender. American Educational Research Journal, 24, 365–385. 10.3102/00028312024003365

[R17] Byrne BM, Shavelson RJ, & Muthén B (1989). Testing for the equivalence of factor covariance and mean structures: The issue of partial measurement invariance. Psychological Bulletin, 105, 456–466. 10.1037/0033-2909.105.3.456

[R18] *Cao X, Wang L, Cao C, Zhang J, & Elhai JD (2017). *DSM-5* posttraumatic stress disorder symptom structure in disaster-exposed adolescents: Stability across gender and relation to behavioral problems. Journal of Abnormal Child Psychology, 45, 803–814. 10.1007/s10802-016-0193-1 27469317

[R19] *Cernvall M, Alaie I, & von Essen L (2011). The factor structure of traumatic stress in parents of children with cancer: A longitudinal analysis. Journal of Pediatric Psychology, 37, 448–457. 10.1093/jpepsy/jsr105 22167122 PMC3334533

[R20] Chen FF (2007). Sensitivity of goodness of fit indexes to lack of measurement invariance. Structural Equation Modeling, 14, 464–504. 10.1080/10705510701301834

[R21] Chen FF (2008). What happens if we compare chopsticks with forks? The impact of making inappropriate comparisons in cross-cultural research. Journal of Personality and Social Psychology, 95, 1005–1018. 10.1037/a0013193 18954190

[R22] Cheung GW, & Rensvold RB (2002). Evaluating goodness-of-fit indexes for testing measurement invariance. Structural Equation Modeling: A Multidisciplinary Journal, 9, 233–255. 10.1207/S15328007SEM0902_5

[R23] Cloitre M, Garvert DW, Weiss B, Carlson EB, & Bryant RA (2014). Distinguishing PTSD, complex PTSD, and borderline personality disorder: A latent class analysis. European Journal of Psychotraumatology, 5, 25097. 10.3402/ejpt.v5.25097 PMC4165723 25279111

[R24] *Contractor AA, Bolton E, Gallagher MW, Rhodes C, Nash WP, & Litz BT (2017). Longitudinal measurement invariance of posttraumatic stress disorder in deployed Marines. Journal of Traumatic Stress, 30, 259–269. 10.1002/jts.22181 28470977

[R25] Contractor AA, Caldas S, Fletcher S, Shea MT, & Armour C (2018). Empirically-derived lifespan polytraumatization typologies: A systematic review. Journal of Clinical Psychology, 1–23. 10.1002/jclp.22586 29363746

[R26] *Contractor AA, Claycomb M, Byllesby B, Layne CM, Kaplow J, Steinberg AM, & Elhai JD (2015). Hispanic ethnicity and Caucasian race: Relations with PTSD’s factor structure in clinic-referred youth. Psychological Trauma: Theory, Research, Practice, and Policy., 7, 456–464. 10.1037/tra0000068 26147448

[R27] Contractor AA, Greene T, Dolan M, & Elhai JD (2018). Relations between PTSD and depression symptom clusters in samples differentiated by PTSD diagnostic status. Journal of Anxiety Disorders, 59, 17–26. 10.1016/j.janxdis.2018.08.004 30142474

[R28] *Contractor AA, Layne CM, Steinberg AM, Ostrowski SA, Ford JD, & Elhai JD (2013). Do gender and age moderate the symptom structure of PTSD? Findings from a national clinical sample of children and adolescents. Psychiatry Research, 210, 1056–1064. 10.1016/j.psychres.2013.09.012 24103907

[R29] Cross MR, & McCanne TR (2001). Validation of a self-report measure of posttraumatic stress disorder in a sample of college-age women. Journal of Traumatic Stress, 14, 135–147. 10.1023/A:1007843800664

[R30] Dimitrov DM (2010). Testing for factorial invariance in the context of construct validation. Measurement and Evaluation in Counseling and Development, 43, 121–149. 10.1177/0748175610373459

[R31] Elhai JD, Biehn TL, Armour C, Klopper JL, Frueh BC, & Palmieri PA (2011). Evidence for a unique PTSD construct represented by PTSD’s D1–D3 symptoms. Journal of Anxiety Disorders, 25, 340–345. 10.1016/j.janxdis.2010.10.007 21094021

[R32] *Elhai JD, Biehn TL, Naifeh JA, & Frueh BC (2011). Posttrau- matic stress disorder instrument wording content is associated with differences in factor structure. Journal of Traumatic Stress, 24, 208–212. 10.1002/jts.20628 21442664

[R33] *Elhai JD, Engdahl R, Palmieri PA, Naifeh JA, Schweinle A, & Jacobs GA (2009). Assessing posttraumatic stress disorder with or without reference to a single, worst traumatic event: Examining differences in factor structure. Psychological Assessment, 21, 629–634. 10.1037/a0016677 19947796

[R34] Elhai JD, & Palmieri PA (2011). The factor structure of posttraumatic stress disorder: A literature update, critique of methodology, and agenda for future research. Journal of Anxiety Disorders, 25, 849–854. 10.1016/j.janxdis.2011.04.007 21793239

[R35] *Elhai JD, Palmieri PA, Biehn TL, Frueh BC, & Magruder KM (2010). Posttraumatic stress disorder’s frequency and intensity ratings are associated with factor structure differences in military veterans. Psychological Assessment, 22, 723–728. 10.1037/a0020643 21038970

[R36] *Engdahl RM, Elhai JD, Richardson JD, & Frueh BC (2011). Comparing posttraumatic stress disorder’s symptom structure between deployed and nondeployed veterans. Psychological Assessment, 23, 1–6. 10.1037/a0020045 21171785

[R37] Foa EB, McLean CP, Zang Y, Rosenfield D, Yadin E, Yarvis JS, … Peterson AL (2018). Effect of prolonged exposure therapy delivered over 2 weeks vs 8 weeks vs present-centered therapy on PTSD symptom severity in military personnel: A randomized clinical trial. JAMA, 319, 354–364. 10.1001/jama.2017.21242 29362795 PMC5833566

[R38] Foa EB, & Tolin DF (2000). Comparison of the PTSD Symptom Scale-Interview version and the Clinician-Administered PTSD Scale. Journal of Traumatic Stress, 13, 181–191. 10.1023/A:1007781909213 10838669

[R39] *Frankfurt S, Armour C, Contractor AA, & Elhai JD (2016). Do gender and directness of trauma exposure moderate PTSD’s latent structure? Psychiatry Research, 245, 365–370. 10.1016/j.psychres.2016.08.049 27591411

[R40] Friedman MJ, Resick PA, Bryant RA, & Brewin CR (2011). Considering PTSD for *DSM-5*. Depression and Anxiety, 28, 750–769. 10.1002/da.20767 21910184

[R41] *Gargurevich R, Luyten P, & Corveleyn J (2009). Factor structure of the Impact of Event Scale-Revised in two different Peruvian samples. Depression and Anxiety, 26, 91–98. 10.1002/da.20430 19180584

[R42] Gregorich SE (2006). Do self-report instruments allow meaningful comparisons across diverse population groups? Testing measurement invariance using the confirmatory factor analysis framework. Medical Care, 44(Suppl. 3), S78–S94. 10.1097/01.mlr.0000245454.12228.8f 17060839 PMC1808350

[R43] Hall BJ, Elhai JD, Grubaugh A, Tuerk P, & Magruder K (2012). Examining the factor structure of PTSD between male and female veterans in primary care. Journal of Anxiety Disorders, 26, 409–415. 10.1016/j.janxdis.2011.12.015 22306134 PMC3610559

[R44] *Hetzel-Riggin MD (2009). A test of structural invariance of posttraumatic stress symptoms in female survivors of sexual and/or physical abuse or assault. Traumatology, 15, 46–59. 10.1177/1534765608331294

[R45] Hoge CW, Auchterlonie JL, & Milliken CS (2006). Mental health problems, use of mental health services, and attrition from military service after returning from deployment to Iraq or Afghanistan. JAMA, 295, 1023–1032. 10.1001/jama.295.9.1023 16507803

[R46] *Hoyt T, & Yeater EA (2010). Comparison of posttraumatic stress disorder symptom structure models in Hispanic and White college students. Psychological Trauma: Theory, Research, Practice, and Policy, 2, 19–30. 10.1037/a0018745

[R47] Hu LT, & Bentler PM (1999). Cutoff criteria for fit indexes in covariance structure analysis: Conventional criteria versus new alternatives. Structural Equation Modeling: A Multidisciplinary Journal, 6, 1–55. 10.1080/10705519909540118

[R48] *Hukkelberg SS (2014). Posttraumatic stress reactions in children and adolescents: Factor structure and gender invariance in the dysphoria and numbing model. Psychological Trauma: Theory, Research, Practice, and Policy, 6, 261–268. 10.1037/a0034463

[R49] Joreskog KG, & Goldberger AS (1975). Estimation of a model with multiple indicators and multiple causes of a single latent variable. Journal of the American Statistical Association, 70, 631—639. 10.1080/01621459.1975.10482485

[R50] *Karstoft KI, Andersen SB, & Nielsen A (2017). Assessing PTSD in the military: Validation of a scale distributed to Danish soldiers after deployment since 1998. Scandinavian Journal of Psychology, 58, 260–268. 10.1111/sjop.12360 28419465

[R51] *Keane TM, Rubin A, Lachowicz M, Brief D, Enggasser JL, Roy M, … Rosenbloom D (2014). Temporal stability of *DSM–5* posttraumatic stress disorder criteria in a problem-drinking sample. Psychological Assessment, 26, 1138–1145. 10.1037/a0037133 24932642 PMC4286303

[R52] Kilpatrick DG, & Saunders BE (1997). Prevalence and consequences of child victimization: Results from the National Survey of Adolescents: Final report. Charleston, SC: National Crime Victims Research and Treatment Center.

[R53] Kim ES, Yoon M, & Lee T (2012). Testing measurement invariance using MIMIC: Likelihood ratio test with a critical value adjustment. Educational and Psychological Measurement, 72, 469–492. 10.1177/0013164411427395

[R54] King DW, Leskin GA, King LA, & Weathers FW (1998). Confirmatory factor analysis of the Clinician-Administered PTSD scale: Evidence for the dimensionality of posttraumatic stress disorder. Psychological Assessment, 10(2), 90–96. 10.1037/1040-3590.10.2.90

[R55] *King DW, Orazem RJ, Lauterbach D, King LA, Hebenstreit CL, & Shalev AY (2009). Factor structure of posttraumatic stress disorder as measured by the Impact of Event Scale–Revised: Stability across cultures and time. Psychological Trauma Theory Research Practice and Policy, 1, 173–187. 10.1037/a0016990

[R56] *Krause ED, Kaltman S, Goodman LA, & Dutton MA (2007). Longitudinal factor structure of posttraumatic stress symptoms related to intimate partner violence. Psychological Assessment, 19, 165–175. 10.1037/1040-3590.19.2.165 17563198

[R57] Little TD, & Slegers DW (Eds.). (2005). Encyclopedia of statistics in behavioral science (Vol. 2). Hoboen, NJ: Wiley.

[R58] Liu P, Wang L, Cao C, Wang R, Zhang J, Zhang B, … Elhai JD (2014). The underlying dimensions of *DSM-5* posttraumatic stress disorder symptoms in an epidemiological sample of Chinese earthquake survivours. Journal of Anxiety Disorders, 28, 345–351. 10.1016/j.janxdis.2014.03.008 24792723

[R59] *Lommen MJ, Van De Schoot R, & Engelhard IM (2014). The experience of traumatic events disrupt the meaurement invariance of a posttraumatic stress scale. Frontiers in Psychology, 5(1304), 1–7. 10.3389/fpsyg.2014.01304 25477835 PMC4235410

[R60] *Mansfield AK, Williams JB, Hourani LL, & Babeu LA (2010). Measurement invariance of posttraumatic stress disorder symptoms among U.S. military personnel. Journal of Traumatic Stress, 23, 91–99. 10.1002/jts.20492 20135678

[R61] *Marshall GN (2004). Posttraumatic Stress Disorder Symptom Checklist: Factor structure and English-Spanish measurement invariance. Journal of Traumatic Stress, 17, 223–230. 10.1023/B:JOTS.0000029265.56982.86 15253094

[R62] *Mason ST, Lauterbach D, McKibben J, Lawrence J, & Fauer- bach JA (2013). Confirmatory factor analysis and invariance of the Davidson Trauma Scale (DTS) in a longitudinal sample of burn patients. Psychological Trauma Theory Research Practice and Policy, 5, 10–17. 10.1037/a0028002

[R63] *McDonald SD, Beckham JC, Morey R, Marx C, Tupler LA, & Calhoun PS (2008). Factorial invariance of posttraumatic stress disorder symptoms across three veteran samples. Journal of Traumatic Stress, 21, 309–317. 10.1002/jts.20344 18553409 PMC2745604

[R64] *Meis LA, Erbes CR, Kaler ME, Arbisi PA, & Polusny MA (2011). The Structure of PTSD among two cohorts of returning soldiers: Before, during, and following deployment to Iraq. Journal of Abnormal Psychology, 120, 807–818. 10.1037/a0023976 21668079

[R65] Meredith W (1993). Measurement invariance, factor analysis and factorial invariance. Psychometrika, 58, 525–543. 10.1007/BF02294825

[R66] Meredith W, & Teresi JA (2006). An essay on measurement and factorial invariance. Medical Care, 44(11, Suppl 3), S69–S77. 10.1097/01.mlr.0000245438.73837.89 17060838

[R67] Miles JN, Marshall GN, & Schell TL (2008). Spanish and English versions of the PTSD Checklist–Civilian version (PCL-C): Testing for differential item functioning. Journal of Traumatic Stress, 21, 369–376. 10.1002/jts.20349 18720394 PMC2958820

[R68] Miller MW, Wolf EJ, Kilpatrick D, Resnick H, Marx BP, Holowka DW, … Friedman MJ (2013). The prevalence and latent structure of proposed *DSM-5* posttraumatic stress disorder symptoms in U.S. national and veteran samples. Psychological Trauma: Theory, Research, Practice, and Policy, 5, 501–512. 10.1037/a0029730

[R69] Millsap RE, & Everson HT (1993). Methodology review: Statistical approaches for assessing measurement bias. Applied Psychological Measurement, 17, 297–334. 10.1177/014662169301700401

[R70] Millsap RE, & Kwok O (2004). Evaluating the impact of partial factorial invariance on selection in two populations. Psychological Methods, 9, 93–115. 10.1037/1082-989X.9.1.93 15053721

[R71] Moher D, Liberati A, Tetzlaff J, Altman DG, & Prisma Group. (2009). Preferred reporting items for systematic reviews and meta-analyses: The PRISMA statement. PLoS med, 6(7), e1000097. 10.7326/0003-4819-151-4-200908180-00135 19621072 PMC2707599

[R72] Newby JH, McCarroll JE, Ursano RJ, Fan Z, Shigemura J, & Tucker-Harris Y (2005). Positive and negative consequences of a military deployment. Military Medicine, 170, 815–819. 10.7205/MILMED.170.10.815 16435750

[R73] Nolte S, Elsworth GR, Sinclair AJ, & Osborne RH (2009). Tests of measurement invariance failed to support the application of the “then- test.” Journal of Clinical Epidemiology, 62, 1173–1180. 10.1016/j.jclinepi.2009.01.021 19595570

[R74] *Nygaard E, Jensen TK, & Dyb G (2012). Stability of posttraumatic stress reaction factors and their relation to general mental health problems in children: A longitudinal study. Journal of Clinical Child and Adolescent Psychology, 41, 15–26. 10.1080/15374416.2012.632344 22233242

[R75] Palmieri PA, Weathers FW, Difede J, & King DW (2007). Confirmatory factor analysis of the PTSD Checklist and the Clinician- Administered PTSD Scale in disaster workers exposed to the World Trade Center Ground Zero. Journal of Abnormal Psychology, 116, 329–341. 10.1037/0021-843X.116.2.329 17516765

[R76] *Pietrzak RH, Feder A, Schechter CB, Singh R, Cancelmo L, Bromet EJ, … Southwick SM (2014). Dimensional structure and course of posttraumatic stress symptomatology in World Trade Center responders. Psychological Medicine, 2, 1–14. 10.1017/S0033291713002924 24289878

[R77] Putnick DL, & Bornstein MH (2016). Measurement invariance conventions and reporting: The state of the art and future directions for psychological research. Developmental Review, 41, 71–90. 10.1016/j.dr.2016.06.004 27942093 PMC5145197

[R78] *Rasmussen A, Verkuilen J, Ho E, & Fan Y (2015). Posttraumatic stress disorder among refugees: Measurement invariance of Harvard Trauma Questionnaire scores across global regions and response patterns. Psychological Assessment, 27, 1160–1170. 10.1037/pas0000115 25894706 PMC4615261

[R79] Resnick HS, Kilpatrick DG, Dansky BS, Saunders BE, & Best CL (1993). Prevalence of civilian trauma and posttraumatic stress disorder in a representative national sample of women. Journal of Consulting and Clinical Psychology, 61, 984–991. 10.1037/0022-006X.61.6.984 8113499

[R80] Rhemtulla M, Brosseau-Liard PE, & Savalei V (2012). When can categorical variables be treated as continuous? A comparison of robust continuous and categorical SEM estimation methods under suboptimal conditions. Psychological Methods, 17, 354–373. 10.1037/a0029315 22799625

[R81] Sack WH, Seeley JR, & Clarke GN (1997). Does PTSD transcend cultural barriers? A study from the Khmer adolescent refugee project. Journal of American Academy of Child and Adolescent Psychiatry, 36, 49–54. 10.1097/00004583-199701000-00017 9000781

[R82] Sass DA (2011). Testing measurement invariance and comparing latent factor means within a confirmatory factor analysis framework. Journal of Psychoeducational Assessment, 29, 347–363. 10.1177/0734282911406661

[R83] *Saul AL, Grant KE, & Carter JS (2008). Post-traumatic reactions in adolescents: How well do the *DSM-IV* PTSD criteria fit the real life experience of trauma exposed youth? Journal of Abnormal Child Psychology, 36, 915–925. 10.1007/s10802-008-9222-z 18330689

[R84] Sheehan DV, Lecrubier Y, Sheehan KH, Amorim P, Janavs J, Weiller E, … Dunbar GC (1998). The Mini International Neuropsychiatric Interview (M.I.N.I.): The development and validation of a structured diagnostic psychiatric interview for *DSM-IV* and *ICD-10*. Journal of Clinical Psychiatry, 59, 22–33. 9881538

[R85] Silverstein MW, Dieujuste N, Kramer LB, Lee DJ, & Weathers FW (2017). Construct validation of the hybrid model of posttraumatic stress disorder: Distinctiveness of the new symptom clusters. Journal of Anxiety Disorders, 54, 17–23. 10.1016/j.janxdis.2017.12.003 29421368

[R86] *Simms LJ, Watson D, & Doebbeling BN (2002). Confirmatory factor analyses of posttraumatic stress symptoms in deployed and nondeployed veterans of the Gulf war. Journal of Abnormal Psychology, 111, 637–647. 10.1037//0021-843X.111.4.637 12428777

[R87] Steinberg AM, Brymer M, Decker K, & Pynoos RS (2004). The UCLA PTSD reaction index. Current Psychiatry Reports, 6, 96–100. 10.1007/s11920-004-0048-2 15038911

[R88] *Sumner JA, Pietrzak RH, Danielson CK, Adams ZW, & Ruggiero KJ (2014). Elucidating dimensions of posttraumatic stress symptoms and their functional correlates in disaster-exposed adolescents. Journal of Psychiatric Research, 59, 85–92. 10.1016/j.jpsychires.2014.09.003 25248557 PMC4252782

[R89] *Suvak M, Maguen S, Litz BT, Silver RC, & Holman EA (2008). Indirect exposure to the September 11 terrorist attacks: Does symptom structure resemble PTSD? Journal of Traumatic Stress, 21, 30–39. 10.1002/jts.20289 18302169

[R90] *Tay AK, Jayasuriya R, Jayasuriya D, & Silove D (2017). Assessing the factorial structure and measurement invariance of PTSD by gender and ethnic groups in Sri Lanka: An analysis of the modified Harvard Trauma Questionnaire (HTQ). Journal of Anxiety Disorders, 47, 45–53. 10.1016/j.janxdis.2017.02.001 28254549

[R91] Tsai J, Harpaz-Rotem I, Armour C, Southwick SM, Krystal JH, & Pietrzak RH (2015). Dimensional structure of *DSM-5* posttraumatic stress symptoms: Results from the National Health and Resilience in Veterans Study. Journal of Clinical Psychiatry, 76, 546–553. 10.4088/JCP.14m09091 25562376

[R92] *Ullman SE, & Long SM (2008). Factor structure of PTSD in a community sample of sexual assault survivors. Journal of Trauma and Dissociation, 9, 507–524. 10.1080/15299730802223370 19042794

[R93] Vandenberg RJ, & Lance CE (2000). A review and synthesis of the measurement invariance literature: Suggestions, practices, and recommendations for organizational research. Organizational Research Methods, 3, 4–70. 10.1177/109442810031002

[R94] *Wang L, Cao X, Cao C, Fang R, Yang H, & Elhai JD (2017). Factor structure of *DSM-5* PTSD symptoms in trauma-exposed adolescents: Examining stability across time. Journal of Anxiety Disorders, 52, 88–94. 10.1016/j.janxdis.2017.07.001 28774745

[R95] *Wang M, Armour C, Li X, Dai X, Zhu X, & Yao S (2013). The factorial invariance across gender of three well-supported models: Further evidence for a five-factor model of posttraumatic stress disorder. Journal of Nervous and Mental Disease, 201, 145–152. 10.1097/NMD.0b013e31827f627d 23364125

[R96] *Wang M, Elhai JD, Dai X, & Yao S (2012). Longitudinal invariance of posttraumatic stress disorder symptoms in adolescent earthquake survivors. Journal of Anxiety Disorders, 26, 263–270. 10.1016/j.janxdis.2011.12.009 22240083

[R97] Weathers FW, Bovin MJ, Lee DJ, Sloan DM, Schnurr PP, Kaloupek DG, … Marx BP (2017). The Clinician-Administered PTSD Scale for *DSM–5* (CAPS-5): Development and initial psychometric evaluation in military veterans. Psychological Assessment, 30(3), 383–395. 10.1037/pas0000486 28493729 PMC5805662

[R98] Weathers FW, Keane TM, & Davidson JRT (2001). Clinician- Administered PTSD Scale: A review of the first 10 years of research. Depression and Anxiety, 13, 132–156. 10.1002/da.1029 11387733

[R99] Weathers FW, Litz BT, Herman DS, Huska JA, & Keane TM (October, 1993). The PTSD Checklist (PCL): Reliability, validity, and diagnostic utility. Paper presented at the International Society for Traumatic Stress Studies, San Antonio, TX.

[R100] Weathers FW, Litz BT, Keane TM, Palmieri PA, Marx BP, & Schnurr PP (2013). The PTSD Checklist for *DSM-5* (PCL-5). Scale available from the National Center for PTSD at www.ptsd.va.gov

[R101] *Wind TR, van der Aa N, de la Rie S, & Knipscheer J (2017). The assessment of psychopathology among traumatized refugees: measurement invariance of the Harvard Trauma Questionnaire and the Hopkins Symptom Checklist-25 across five linguistic groups. European Journal of Psychotraumatology, 8, 1321357. 10.1080/20008198.2017.1321357 29038686 PMC5632793

[R102] Wu AD, Li Z, & Zumbo BD (2007). Decoding the meaning of factorial invariance and updating the practice of multi-group confirmatory factor analysis: A demonstration with TIMSS data. Practical Assessment, Research & Evaluation, 12, 1–26.

[R103] Yuan KH, & Chan W (2016). Measurement invariance via multigroup SEM: Issues and solutions with chi-square-difference tests. Psychological Methods, 21, 405–426. 10.1037/met0000080 27266799

[R104] *Zelazny K, & Simms LJ (2015). Confirmatory factor analyses of *DSM-5* posttraumatic stress disorder symptoms in psychiatric samples differing in Criterion A status. Journal of Anxiety Disorders, 34, 15–23. 10.1016/j.janxdis.2015.05.009 26103594

