# Supplementary material for: Invariance of the Construct of Posttraumatic Stress Disorder: A Systematic Review
Source: J Trauma Stress. Author manuscript; Available in PMC 2026 Apr 29. (PMC13126149; doi:10.1002/jts.22389)
Supplement: Supplemental Material [file NIHMS2164437-supplement-Supplemental_Material.docx]

Supplemental Table 1. *Descriptive information of studies included in the review*

| **Study** | **Type of Sample** | **Gender %** | **Race/Ethnicity %** | **Nature of subgroups** |
| --- | --- | --- | --- | --- |
| **PTSD Checklist (PCL)** | | | | |
| Asmundson et al. (2003) | -Canada (*n* = 787)  -Regular/reserve United Nations peacekeepers deployed overseas (*M*_age_ = 48.80-49.90; *SD*_age_ = 10.40-11.50) | -Male: 787 (100%)  -Female: 0 (0%) | -NR | -Chronic back pain vs. No chronic back pain |
| Biehn et al. (2012) | -Canada (*n* = 378)  -Veterans (*M*_age_ = 48.70; *SD*_age_ = 16.67) | -Male: 372 (95.6%)  -Female: 18 (4.4%) | -NR | -PTSD vs. No PTSD |
| Boal et al. (2017) | -US (*n* = 455)  -Airmen veterans (*M*_age_ = 36.40; *SD*_age_ = 9.08) | -Male: 391 (85.9%)  -Female: 64 (16.4%) | -White: 364 (79.9%)  -Hispanic: 49 (10.8%)  -Black: 31 (6.8%)  -Other: 11 (2.5%) | -Phone interview vs. Web |
| Cao et al. (2017) | -China (*n* = 1,184)  -Students in a town that experienced an earthquake disaster (*M*_age_ = 14.30; *SD*_age_ = 0.80) | -Male: 547 (46.2%)  -Female: 637 (58.3%) | -Qiang: 771 (65.1%)  -Han: 385 (32.5%)  -Other ethnicities in China: 28 (2.3%) | -Boys vs. Girls |
| Cernvall et al. (2011) | -Sweden (*n* = 249)  -Parents of children receiving treatment for cancer (*M*_age_ = 37.00-40.00; *SD*_age_ = 6.30-6.80) | -Male: 121 (48.6%)  -Female: 128 (51.4%) | -NR | -Time 1 (2 weeks post-cancer diagnosis) vs. Time 2 (2 months post-cancer diagnosis) vs. Time 3 (4 months post-cancer diagnosis) |
| Elhai et al. (2009) | -US (*n* = 916)  -Undergraduate students (*M*_age_ = 20.43; *SD*_age_ = 3.86) | -Male: 302 (33.0%)  -Female: 614 (67.0%) | -Caucasian: 871 (95.4%)  -Hispanic/Latino: 16 (1.8%) | -Trauma-specific group vs. No trauma group  -Trauma-general group vs. No-trauma group  -Trauma-specific group vs. Trauma-general group |
| Engdahl et al. (2011) | -Canada (*n* = 1,066)  -Veterans receiving/eligible for disability pension (*M*_age_ = 45.33; *SD*_age_ = 10.05) | -Male: 981 (92.0%)  -Female: 85 (8.0%) | -NR | -Non-deployed vs. Deployed |
| Frankfurt et al. (2016) | -US (*n* = 455)  -University students (*M*_age_ = 20.07; *SD*_age_ = 4.41) | -Male: 149 (33.7%)  -Female: 309 (67.3%) | -NR | -Males vs. Females  -Direct trauma exposure vs. Indirect trauma exposure |
| Hoyt et al. (2010) | -US (*n* = 504)  -Undergraduate students (*M*_age_ = 19.90; *SD*_age_ = 6.50) | -Male: 160 (31.7%)  -Female: 344 (68.3%) | -White: 278 (55.2%)  -Hispanic: 226 (44.8%) | -Hispanic vs. White |
| Keane et al. (2014) | -US (*n* = 507)  -Veterans of Iraq and Afghanistan in an online intervention for problem drinking/combat-related stress (*M*_age_ = 31.90; *SD*_age_ = 7.70) | -Male: 442 (87.2%)  -Female: 65 (12.8%) | -White: 405 (79.9%) | -Baseline vs. 8-week post-intervention vs. 3-month follow up |
| Krause et al. (2007) | -US (*n* = 801)  -Low-income women who experienced IPV and receiving medical treatment (IPV sample) or who were receiving IPV-related treatment (IPV-services; *M*_age_ = 31.00-33.00; *SD*_age_ = 8.59-10.16) | -Male: 0 (0%)  -Female: 801 (100%) | -African American (81.0%-95.0%)  -Latina (1.0%)  -Other groups (4.0%-5.0%) | -IPV sample vs. IPV-services  -IPV-services: Time 1 (approximately 1 month post-IPV) vs. Time 2 (approximately 3 months post-IPV) |
| Mansfield et al. (2010) | -US (*n* = 15,593)  -Active duty personnel from a population-based study (*M*_age_ = 28.40; *SD*_age_ = 0.30) | -Male: 11,710 (75.1%)  -Female: 3,883 (24.9%) | -White, non-Hispanic: 10,089 (64.7%) | -Non-deployed vs. Deployed |
| Marshall (2004) | -US (*n* = 419)  -Individuals from a trauma facility for treatment of injuries from community violence (*M*_age_ = 24.30; *SD*_age_ = 5.60) | -Male: 394 (94.0%)  -Female: 25 (6.0%) | -Hispanic: 327 (78.0%)  -Black: 50 (12.0%)  -Non-Hispanic Caucasian: 13 (3.0%)  -Asian: 17 (4.0%)  -Native American, multiracial, other: 17 (4.0%) | -English vs. Spanish |
| Meis et al. (2011) | -US (*n* = 2,965)  -Two samples from an Army National Guard Brigade Combat Team who conducted an extended deployment to Iraq (*M*_age_ = 29.90-31.50; *SD*_age_ = 7.96-8.72) | -Male: 2,621-2,760 (88.4%-93.1%)  -Female: 181-344 (6.1%-11.6%) | -White: 2,538-2,781 (85.6%-93.8%)  -Black: 68-148 (2.3%-5.0%)  -Latino/a: 62-160 (2.1%-5.4%)  -Native American: 24-77 (0.1%-2.6%)  -Asian: 47-56 (1.6%-1.9%) | -Sample 1 at Time 3 (15 months post-return) vs. Sample 2 at Time 2 (12 months post-return)  -Sample 1: Time 1 (1 month pre-deployment) vs. Time 2 (2-3 months post-deployment) vs. Time 3 (15 months post-deployment)  -Sample 2: Time 1 (during deployment) vs. Time 2 (12 months post-deployment) |
| Pietrzak et al. (2014) | -US (*n* = 10,835)  -World Trade Center responders, including a sample of police and a sample of non-traditional responders (e.g., construction workers and utility workers; *M*_age_ = 41.20-45.30; *SD*_age_ = 6.60-9.60) | -Male: 3,443-5,886 (85.3%-86.7%)  -Female: 592-913 (13.4%-14.7%) | -White: 2,732-4,145 (61.0%-67.4%)  -Black: 396-808 (9.8%-11.9%)  -Hispanic: 817-1,663 (20.3%-24.5%)  -Other: 88-182 (2.2%-2.7%) | -Police responders: Time 1 (average 3.30 years post-9/11) vs. Time 2 (average 5.70 years post-9/11) vs. Time 3 (average 5.30 years post-9/11)  -Non-traditional responders: Time 1 (average 3.30 years post-9/11) vs. Time 2 (average 5.70 years post-9/11) vs. Time 3 (average 5.30 years post-9/11) |
| Simms et al. (2002) | -US (*n* = 3,695)  -Active duty or activated National Guard or US Army Reserve during Gulf War (*M*_age_ and *SD*_age_: NR) | -Male: 3,362 (91.0%)  -Female: 333 (9.0%) | -Caucasian: 3,547 (96.0%) | -Deployed Sample 1 vs. Deployed Sample 2  -Deployment history vs. No deployment history |
| Wang et al. (2012) | -China (*n* = 403)  -Middle school student earthquake survivors (*M*_age_ = 14.00; *SD*_age_ = 1.50) | -Male: 189 (33.3%)  -Female: 214 (53.1%) | -NR | -Time 1 (5 months post-earthquake) vs. Time 2 (11 months post-earthquake) |
| Wang et al. (2013) | -China (*n* = 571)  -Children and adolescents earthquake survivors (*M*_age_ = 14.00; *SD*_age_ = 1.16) | -Male: 297 (52.0%)  -Female: 274 (48.0%) | -NR | -Boys vs. Girls |
| Wang et al. (2017) | -China (*n* = 836)  -Students near a blast site of a series of chemical explosions (*M*_age_ = 12.50; *SD*_age_ = 2.30) | -Male: 429 (51.3%)  -Female: 407 (48.7%) | -Han: 791 (94.6%)  -Other: 37 (4.4%) | -Wave 1 (3 months post-blast) vs. Wave 2 (8 months post-blast) |
| **PCL and Clinician-Administered PTSD Scale (CAPS)** | | | | |
| Contractor et al. (2017) | -US (*n* = 834)  -Active duty combat Marines (*M*_age_ = 23.20; *SD*_age_ = 3.70) | -Male: 834 (100%)  -Female: 0 (0%) | -Caucasian: 685 (82.9%)  -Black or African American: 44 (5.4%)  -American Indian: 12 (1.4%)  -Asian: 24 (2.9%)  -Native Hawaiian/Pacific Islander: 17 (2.0%) | -PCL: Time 0 (1 month pre-deployment) vs. Time 1 (1 month post-deployment) vs. Time 2 (5 months post-deployment) vs. Time 3 (8 months post-deployment)  -CAPS: Time 0 vs. Time 2 vs. Time 3 |
| **PCL and PTSD Symptom Scale (PSS)** | | | | |
| Elhai et al. (2011) | -US (*n* = 385)  -Undergraduate students (*M*_age_ = 19.96; *SD*_age_ = 4.44) | -Male: 117 (30.4%)  -Female: 268 (69.6%) | -Caucasian: 281 (73.0%)  -African American: 83 (21.6%)  -Hispanic/Latino: 69 (17.9%) | -PCL-S vs. PSS |
| **UCLA PTSD Reaction Index-Revised (PTSD-RI)** | | | | |
| Armour et al. (2011a) | -Bosnia (*n* = 1,362)  -War-exposed secondary students (*M*_age_ = 16.20; *SD*_age_ = 1.60) | -Male: 416 (30.5%)  -Female: 919 (67.5%) | -NR | -Criterion A2 group vs. Non-Criterion A2 group |
| Armour et al. (2011b) | -Bosnia (*n* = 1,480)  -War-exposed secondary students (*M*_age_ = 16.18; *SD*_age_ = 1.07) | -Male: 471 (31.8%)  -Female: 1,009 (68.2%) | -NR | -Boys vs. Girls |
| Bennett et al. (2014) | -US (*n* = 1,363)  -Youth recruited from two juvenile detention centers in the West and Midwest (*M*_age_ = 15.60; *SD*_age_ = 1.41) | -Male: 990 (72.6%)  -Female: 373 (27.4%) | -White/Caucasian: 885 (64.9%)  -Black/African  American: 263 (19.3%)  -Hispanic/Latino: 119 (8.7%)  -Multiracial: 42 (3.1%)  -Other: 45 (3.3%) | -Boys vs. Girls |
| Contractor et al. (2013) | -US (*n* = 6,591)  -Children and adolescents presenting for mental health care services (*M*_age_ = 12.64; *SD*_age_ = 3.08) | -Male: 2,934 (44.5%)  -Female: 3,657 (55.5%) | -Caucasian: 3,767 (57.2%)  -African American: 1,812 (27.5%)  -Hispanic: 2,395 (38.3%) | -Pre-adolescents vs. Adolescents  -Males vs. Females |
| Contractor et al. (2015) | -US (*n* = 6,248-6,591)  -Children and adolescents presenting for mental health care services (*M*_age_ = 12.70-12.64; *SD*_age_ = 3.09-3.08) | -Male: 2,785-2,934 (44.5%-44.5%)  -Female: 3,463-3,657 (55.4%-55.5%) | -Caucasian: 3,618-3,767 (57.2%-57.9%)  -African American: 1,698-1,812 (27.2%-27.5%)  -Hispanic ethnicity: 2,395 (38.3%) | -Hispanics vs. Non-Hispanics  -Caucasians vs. Non-Caucasians |
| Nygaard et al. (2012) | -Norway (*n* = 133)  -Norwegian children who were in affected areas during a tsunami in Southeast Asia (*M*_age_ = 12.90; *SD*_age_ = 3.40) | -Male: 61 (45.9%)  -Female: 72 (54.1%) | -NR | -Time 1 (10-11 months post–tsunami) vs. Time 2 (2.5 years post–tsunami) |
| **Impact of Event Scale–Revised (IES-R)** | | | | |
| Gargurevich et al. (2009) | -Peru (*n* = 736)  -Survivors of a village fire and a university student sample (*M*_age_ = 21.00-29.00; *SD*_age_ = 4.30-7.80) | -Male: 295 (40.1%)  -Female: 441 (59.9%) | -NR | -Fire survivors vs. University students |
| King et al. (2009) | -Israel and US (*n* = 541)  -Emergency room patients (Israel) and undergraduate students (US; *M*_age_ = 21.76-31.57; *SD*_age_ = 5.96-10.72) | -Male: 214 (39.6%)  -Female: 327 (60.4%) | US sample only:  -European American (65.0%)  -African America (32.0%)  -Other (3.0%) | -US students vs. Israeli ER patients at Time 1 (average 9.90 days post-ER admission)  -Israel patients: Time 1 (average 9.90 days post-ER admission) vs. Time 2 (average 39.80 days post-ER admission) vs. T3 (average 168.40 days post-ER admission) |
| Suvak et al. (2008) | -US (*n* = 685)  -Sample of individuals invited to complete a web-based survey (*M*_age_ = 47.30; *SD*_age_ = 16.50) | -Male: 340 (50.4%)  -Female: 335 (49.6%) | -White: 552 (86.1%)  -African American: 55 (8.6%)  -Native American: 8 (1.3%)  -Asian: 9 (1.4%)  -Other: 17 (2.7%) | -Wave 2 (approximately 2 months post-9/11) vs. Wave 3 (approximately 6 months post-9/11) |
| **Harvard Trauma Questionnaire (HTQ)** | | | | |
| Rasmussen et al. (2015) | -US (*n* = 878)  -Survivors of torture/human rights  abuses from different regions around the world (*M*_age_ = 34.90; *SD*_age_ = 9.92) | -Male: 518 (59.0%)  -Female: 360 (41.0%) | -West African origin: 306 (34.9%)  -Himalayan Asia origin: 188 (21.4%)  -Central African origin: 383 (14.0%) | -West Africans vs. Himalayans vs. Others |
| Tay et al. (2017) | -Sri Lanka (*n* = 4,260)  -Part of a representative survey conducted across Sri Lanka (*M*_age_ = 43.00; *SD*_age_ = 13.86) | -Male: 1,223 (28.7%)  -Female: 3,037 (71.3%) | -Sinhalese (31.1%)  -Tamil (45.8%)  -Moors (23.1%) | -Males vs. Females  -Sinhalese vs. Tamil vs. Moor |
| Wind et al. (2017) | -Netherlands (*n* = 1,256)  -Dutch and refugee patients referred  to a center for treatment/diagnosis of  complex trauma (*M*_age_ = 43.30; *SD*_age_ = 11.00) | -Male: 892 (71.0%)  -Female: 364 (29.0%) | -Indo-Iranian: 262 (20.9%)  -Niger-Congo: 134 (10.7%)  -Semitic: 288 (22.9%)  -South Slavic: 199 (15.8%)  -Germanic: 373 (29.7%) | -Indo-Iranian language vs. Niger-Congo language vs. Semitic language vs. South Slavic language vs. Germanic language |
| **Posttraumatic Stress Diagnostic Scale (PDS)** | | | | |
| Baschnagel et al. (2005) | -US (*n* = 528)  -Undergraduate students (*M*_age_ = 19.50; *SD*_age_ = 3.20) | -Male: 238 (45.1%)  -Female: 290 (54.9%) | -Caucasian: 380 (72.0%)  -Asian: 58 (11.0%)  -African American: 48 (9.1%) | -Time 1 (1 month post-9/11 attack) vs. Time 2 (3 months post-9/11 attack) |
| Ullman et al. (2008) | -US (*n* = 967)  -Adult female sexual assault survivors (*M*_age_ = 32.00; *SD*_age_: NR) | -Male: 0 (0%)  -Female: 967 (100%) | -African American (43.2%)  -White (39.0%)  -Asian (2.5%)  -Mixed racial identity (6.9%)  -Hispanic (6.4%)  -Other (0.9%) | -Black vs. White  -High school or less vs. Some college and beyond |
| **Child PTSD Symptom Scale (CPSS)** | | | | |
| Hukkelberg (2014) | -Norway (*n* = 390)  -Children and adolescents receiving services from child guidance clinics (*M*_age_ = 14.26; *SD*_age_ = 2.32) | -Male: 152 (39.0%)  -Female: 238 (61.0%) | -Norwegian: 291 (74.6%)  -One Norwegian parent: 31 (7.9%)  -Scandinavian origin: 3 (0.8%)  -Asian: 36 (9.2%)  -African: 12 (3.1%)  -From South or Central America: 3 (0.8%)  -Other: 3 (0.8%) | -Boys vs. Girls |
| **PTSD Symptom Scale (PSS)** | | | | |
| Lommen et al. (2014) | -Denmark (*n* = 554)  -Soldiers before and after deployment to Afghanistan and Iraq (*M*_age_ and *SD*_age_: NR) | -NR | -NR | -Dutch sample 1 – Afghanistan deployment: Time 1 (2 months pre-deployment) vs. Time 2 (2 months post-deployment)  -Dutch sample 2 – Iraq deployment; Time 1 (pre-deployment) vs. Time 2 (5 months post-deployment)  -Deployment history: with vs. without |
| **Davidson Trauma Scale (DTS)** | | | | |
| Mason et al. (2013) | -US (*n* = 299)  -Adult patients admitted to a regional burn center for treatment of burn injury (*M*_age_ = 39.16; *SD*_age_ = 15.66) | -Male: 207 (62.9%)  -Female: 92 (30.8%) | -Caucasian American: 181 (60.7%) | -1 month post-baseline assessment vs. 6 month post-baseline assessment vs. 12 month post-baseline assessment vs. 24 month post-baseline assessment |
| McDonald et al. (2008) | -US (*n* = 1,440)  -Three groups of military veterans who differ in era of military service (Range_age_ = 18-74) | -Male: 1,355 (94.1%)  -Female: 86 (5.9%) | -Caucasian: 605 (42.0%)  -African American: 724 (50.3%)  -Other/unknown: 82 (5.7%) | -OEF/OIF veteran research participants vs. Post-Vietnam veterans  -OEF/OIF veteran research participants vs. Vietnam veterans  -Post-Vietnam veterans vs. Vietnam veterans |
| **Clinician-Administered PTSD Scale (CAPS)** | | | | |
| Elhai et al. (2010) | -US (*n* = 747)  -Veterans from VA Medical Centers’ primary care clinics (*M*_age_ = 61.20; *SD*_age_ = 11.80) | -Male: 696 (93.2%)  -Female: 51 (6.8%) | -Caucasian: 468 (62.7%)  -African American: 259 (34.7%) | -PTSD frequency format vs. PTSD intensity format |
| **Posttraumatic Stress Disorder Questionnaire (PTSD-Q)** | | | | |
| Hetzel-Riggin (2009) | -US (*n* = 2,378)  -Undergraduate students with reported history of physical and/or sexual abuse (*M*_age_ = 18.90; *SD*_age_ = 2.60) | -Male: 0 (0%)  -Female: 2,378 (100%) | -Caucasian 2,038 (85.7%)  -African America: 697 (29.3%)  -Latino/a: 213 (9.0%)  -Asian: 189 (7.9%)  -Other: 83 (3.5%) | -Child sexual abuse vs. Child physical abuse vs. Adult sexual assault vs. Adult physical assault vs. Multiple abuse |
| **Psychological Reactions Following International Missions Questionnaire (PRIM)** | | | | |
| Karstoft et al. (2017) | -Netherlands (*n* = 612)  -Soldiers deployed to Afghanistan (*M*_age_ and *SD*_age_: NR) | -NR | -NR | -Cohort 1 (returning from deployment in 2009) vs. Cohort 2 (returning from deployment in 2013) |
| **Diagnostic Interview Schedule – PTSD Module** | | | | |
| Saul et al. (2008) | -US (*n* = 1,581)  -Trauma- exposed youth from the National Survey of Adolescents (Range_age_ = 12-17) | -Male: 824 (52.1%)  -Female: 757 (47.9%) | -Latino 153 (9.7%)  -Asian: 27 (1.7%) | -Males vs. Females  -Children ages 12-14 vs. 15-17  -Violent vs. Non-violent traumatic stressors |
| **National Survey of Adolescents – PTSD Module** | | | | |
| Sumner et al. (2014) | -US (*n* = 1,999)  -Tornado-exposed adolescents (*M*_age_ = 14.50; *SD*_age_ = 1.70) | -Male: 980 (49.0%)  -Female: 1,019 (51.0%) | -White: 1,407 (70.4%)  -Black: 508 (25.4%)  -Other: 84 (4.2%) | -Males vs. Females  -Children ages 12-14 vs. Children ages 15-17 |
| **Mini-International Neuropsychiatric Interview 6 (MINI-6) – PTSD Module** | | | | |
| Zelazny et al. (2015) | -US (*n* = 594*)  -Adults in treatment with a mental health practitioner currently or at some point in the last two years (*M*_age_ = 43.20; *SD*_age_ = 12.50) | -Male: 226 (36.0%)  -Female 402 (64.0%) | -Caucasian: 396 (63.0%)  -African American: 214 (34.0%)  -American Indian or Alaskan Native: 13 (2.0%)  -Asian: 3 (0.5%)  -Native Hawaiian or Other Pacific Islander: 1 (0.2%) | -Criterion A vs. Non-criterion A stressful life event |
| *Note*. NR is not reported; IPV is intimate partner violence; VA is Veteran Affairs; *Demographic data is based on an initial sample of 628 participants, but analyses only involved 594 participants who completed measures of interest. | | | | |

Supplemental Table 2. *Item mappings for DSM-IV PTSD factor-analytical models*

| **DSM-IV PTSD symptoms** | **Taylor et. al.** | **3-factor** | **Smith et al.** | **Modified 3-factor** | **4-factor with sleep** | **EN** | **Dysphoria** | **DA (m)** |
| --- | --- | --- | --- | --- | --- | --- | --- | --- |
| B1. Intrusive thoughts | I-Av | I | I | I | I | I | I | I |
| B2. Nightmares | I-Av | I | I | I | S/I | I (S)* | I (S)* | I |
| B3. Reliving traumas | I-Av | I | Av | I | I | I | I | I |
| B4. Emotional cue reactivity | I-Av | I | Av | I | I | I | I | I |
| B5. Physiological cue reactivity | I-Av | I | Av | I | I/H | I | I | I |
| C1. Avoidance of thoughts | I-Av | Av-N | Av | Av | Av-N or Av | Av | Av | Av |
| C2. Avoidance of Reminders | I-Av | Av-N | Av | Av | Av-N or Av | Av | Av | Av |
| C3. Amnesia for traumatic event | N-H | Av-N | Av | Av | Av-N or Av | N | D | N |
| C4. Loss of interest | N-H | Av-N | N | H | - | N | D | N |
| C5. Detachment | N-H | Av-N | N | H | - | N | D | N |
| C6. Restricted affect | N-H | Av-N | N | H | Av-N or Av | N | D | N |
| C7. Hopelessness | N-H | Av-N | N | H | - | N | D | N |
| D1. Sleeping difficulties | N-H | H | H | I | S | H (S)* | D (S)* | DA |
| D2. Irritability/anger | N-H | H | N | H | H | H | D | DA |
| D3. Concentration difficulties | N-H | H | H | H | H | H | D | DA |
| D4. Hypervigilance | N-H | H | - | H | H | H | H | AA |
| D5. Startled easily | N-H | H | - | H | H | H | H | AA |

*Note*. EN is Emotional Numbing Model; DA (m) is Dysphoric Arousal Model; I is intrusions; Av is avoidance; N is numbing; H is hyperarousal; D is dysphoria; DA is dysphoric arousal; AA is anxious arousal; S/(S)* indicates that the particular item loaded on the sleep factor (King et al., 2009); Modified 3-factor model used in the Wind et al. (2017) study; 4-factor with sleep model used in Gargurevich et al. (2009) and King et al. (2009) studies.

| Supplemental Table 3. *Item mappings for DSM-5 PTSD factor-analytic models* | | | | |  |  | |  |  | |  | |  |
| --- | --- | --- | --- | --- | --- | --- | --- | --- | --- | --- | --- | --- | --- |
| **DSM-5 PTSD Symptoms** | **DSM-5** | **Modified DSM-5** | **Dysphoria** | **DA** | | | **Anhedonia** | | | **Externalizing** | | **Hybrid** | |
| 1. Intrusive thoughts | I | I | I | I | | | I | | | I | | I | |
| 2. Recurrent Nightmares | I | I | I | I | | | I | | | I | | I | |
| 3. Flashbacks | I | I | I | I | | | I | | | I | | I | |
| 4. Emotional reactivity | I | I | I | I | | | I | | | I | | I | |
| 5. Physiological reactivity | I | I | I | I | | | I | | | I | | I | |
| 6. Avoidance of thoughts | Av | AN | Av | Av | | | Av | | | Av | | Av | |
| 7. Avoidance of reminders | Av | AN | Av | Av | | | Av | | | Av | | Av | |
| 8. Memory impairment | NACM | AN | D | NACM | | | NACM | | | NACM | | NA | |
| 9. Negative beliefs | NACM | NACM | D | NACM | | | NACM | | | NACM | | NA | |
| 10. Blame of self or others | NACM | NACM | D | NACM | | | NACM | | | NACM | | NA | |
| 11. Negative TE-related emotions | NACM | NACM | D | NACM | | | NACM | | | NACM | | NA | |
| 12. Loss of interest | NACM | AN | D | NACM | | | Anh | | | NACM | | Anh | |
| 13. Detachment | NACM | AN | D | NACM | | | Anh | | | NACM | | Anh | |
| 14. Restricted range of affect | NACM | NACM | D | NACM | | | Anh | | | NACM | | Anh | |
| 15. Irritability/anger | AAR | - | D | DA | | | DA | | | EB | | EB | |
| 16. Risky or destructive behavior | AAR | - | D | DA | | | DA | | | EB | | EB | |
| 17. Hypervigilance | AAR | HYP | AAR | AA | | | AA | | | AA | | AA | |
| 18. Exaggerated startle response | AAR | HYP | AAR | AA | | | AA | | | AA | | AA | |
| 19. Difficulty concentrating | AAR | HYP | D | DA | | | DA | | | DA | | DA | |
| 20. Difficulty sleeping | AAR | HYP | D | DA | | | DA | | | DA | | DA | |

*Note*. I is intrusion; Av is avoidance; NACM is negative alterations in cognitions and mood; AAR is alterations in arousal and reactivity; NA is negative affect; Anh is anhedonia; EB is externalizing behaviors; AA is anxious arousal; D is dysphoria; DA is dysphoric arousal; HYP is hyperarousal; AN is avoidance/numbing; Modified DSM-5 model used in Tay et al. (2017) with items mapped approximately.

Supplemental Table 4. *Aspects related to* *invariance testing*

| **PTSD Model** | **PTSD Scale** | **Invariance Models Examined; Estimator** | **Fit indices for Each Model** | **Invariance Model Comparison Criteria (∆)** | **Invariance Models** | **Partial Invariance Results** |
| --- | --- | --- | --- | --- | --- | --- |
| **PTSD Checklist (PCL)** | | | | | | |
| Asmundson et al. (2003); CS; Chronic back pain (*n*=427; 54.3%) vs. No chronic back pain (*n*=341; 43.3%)^j^ | | | | | | |
| EN | PCL-M (*DSM-IV-TR*)  -Self-report | A, B; NR | S-Bχ^2^, CFI-R | ∆S-Bχ^2^ | A | Metric partial invariance by releasing factor loading constraints on I(a), N(b), N(c), and H(d) |
| Hierarchical Taylor et al. 2-factor | PCL-M (*DSM-IV-TR*)  -Self-report | A, B; NR | S-Bχ^2^, CFI-R | ∆S-Bχ^2^ | A | Metric partial invariance by releasing factor loading constraints on I/Av(a), N-H(b), and N/H(c) |
| Biehn et al. (2012); CS; PTSD (*n*=230; 60.8%) vs. No PTSD (*n=*148; 39.2%)^j^ | | | | | | |
| Dysphoria | PCL-M (*DSM-IV*)  -Self-report | A-F; ML | χ^2^ | ∆χ^2^ | A, B | - |
| EN | PCL-M (*DSM-IV*)  -Self-report | A-F; ML | χ^2^ | ∆χ^2^ | A, B | - |
| Boal et al. (2017); CS; Phone interview (*n*=299; 65.7%) vs. Web (*n*=156; 43.3%)^g^ | | | | | | |
| Dysphoria | PCL (*DSM-IV*)  -Interview  -Self-report | A-C; MLR | S-Bχ^2^, RMSEA, CFI, AIC | ∆S-Bχ^2^ | A, B | Partial scalar invariance by releasing intercept constraints on D(e), D(f), and D(g) |
| Cao et al. (2017); CS; Boys (*n*=547; 46.2%) vs. Girls *(n*=637; 58.3%)^a^ | | | | | | |
| Hybrid | PCL-5 (*DSM-5*)  -Self-report | A-F (Indices reported for Models A-E only); ML | S-Bχ^2^, RMSEA, CFI, TLI, BIC | ∆CFI | A-E | - |
| Cernvall et al. (2011); Longitudinal; T1 (2 weeks post-cancer diagnosis; *n*=249; 100%) vs. T2 (2 months post-cancer diagnosis; *n*=234; 94.0%) vs. T3 (4 months post-cancer diagnosis; *n*=203; 81.5%)^e^ | | | | | | |
| 3-Factor *DSM-IV* | PCL-C (*DSM-IV*)  -Interview (phone) | A, B, E (Phi); MLR | MLR χ^2^, RMSEA, CFI, BIC | ∆S-Bχ^2^, ∆CFI | A | - |
| Dysphoria | PCL-C (*DSM-IV*)  -Interview (phone) | A, B, E (Phi); MLR | MLR χ^2^, RMSEA, CFI, BIC | ∆S-Bχ^2^, ∆CFI | A, B | - |
| EN | PCL-C (*DSM-IV*)  -Interview (phone) | A, B, E (Phi); MLR | MLR χ^2^, RMSEA, CFI, BIC | ∆S-Bχ^2^, ∆CFI | A, B | - |
| Contractor et al. (2017); Longitudinal; PCL; T0 (1 month pre-deployment; *n*=834; 100%) vs. T1 (1 month post-deployment; *n*=531; 63.7%) vs. T2 (5 months post-deployment; *n*=312; 37.4%) vs. T3 (8 months post-deployment; *n*=273; 32.7%)^h^ | | | | | | |
| EN (parceled) | PCL (*DSM-IV*)  -Self-report | A-C; MLR | χ^2^, RMSEA, CFI, TLI, SRMR, BIC | ∆χ^2^, ∆CFI | A | Partial metric invariance by releasing factor-loading constraints on I(a), and N(e); Partial scalar invariance by releasing intercept and factor loadings constraints on N(c) |
| 1-Factor | PCL (*DSM-IV*)  -Self-report | A-C; MLR | χ^2^, RMSEA, CFI, TLI, SRMR, BIC | ∆χ^2^, ∆CFI | A, B | Scalar invariance by releasing factor loading and intercept constraints on N and Av |
| Elhai et al. (2009); CS; Trauma-specific group (*n*=218; 23.8%) vs. No-trauma group (*n*=464; 50.7%)^d^ | | | | | | |
| EN | PCL-S (*DSM-IV*)  -Self-report | A-F; MLM | S-Bχ^2^ | ∆S-Bχ^2^ | A | - |
| Dysphoria | PCL-S (*DSM-IV*)  -Self-report | A-F; MLM | S-Bχ^2^ | ∆S-Bχ^2^ | A | - |
| Elhai et al. (2009); CS; Trauma-general group (*n*=234; 25.5%) vs. No-trauma group (*n*=464; 50.7%)^d^ | | | | | | |
| EN | PCL-S (*DSM-IV*)  -Self-report | A-F; MLM | S-Bχ^2^ | ∆S-Bχ^2^ | A, B | - |
| Dysphoria | PCL-S (*DSM-IV*)  -Self-report | A-F; MLM | S-Bχ^2^ | ∆S-Bχ^2^ | A, B | - |
| Elhai et al. (2009); CS; Trauma-specific group (*n*=218; 23.8%) vs. Trauma-general group (*n*=234; 25.5%)^d^ | | | | | | |
| EN | PCL-S (*DSM-IV*)  -Self-report | A-F; MLM | S-Bχ^2^ | ∆S-Bχ^2^ | A-D, F | - |
| Dysphoria | PCL-S (*DSM-IV*)  -Self-report | A-F; MLM | S-Bχ^2^ | ∆S-Bχ^2^ | A-D, F | - |
| Engdahl et al. (2011); CS; Non-deployed (*n*=320; 30.1%) vs. Deployed (*n*=742; 69.9%)^f^ | | | | | | |
| EN | PCL-M (*DSM-IV-TR*)  -Self-report | A-F; MLM | S-Bχ^2^ | ∆S-Bχ^2^ | A, C | - |
| Dysphoria | PCL-M (*DSM-IV-TR*)  -Self-report | A-F; MLM | S-Bχ^2^ | ∆S-Bχ^2^ | A, C | - |
| Frankfurt et al. (2016); CS; Males (*n*=149; 33.7%) vs. Females (*n*=306; 67.3%)^a^ | | | | | | |
| DSM-5 | PCL-5 (*DSM-5*)  -Self-report | A; MLR | χ^2^, RMSEA, CFI, TLI, BIC (A only) | ∆χ^2^, ∆CFI | -No Invariance | - |
| EB | PCL-5 *(DSM-5*)  -Self-report | A; MLR | χ^2^, RMSEA, CFI, TLI, BIC (A only) | ∆χ^2^, ∆CFI | -No Invariance | - |
| Anh | PCL-5 (*DSM-5*)  -Self-report | A-C; MLR | χ^2^, RMSEA, CFI, TLI, BIC (A only) | ∆χ^2^, ∆CFI | A-C | - |
| Hybrid | PCL-5 (*DSM-5*)  -Self-report | A-C; MLR | χ^2^, RMSEA, CFI, TLI, BIC (A only) | ∆χ^2^, ∆CFI | A-C | - |
| Frankfurt et al. (2016); CS; Direct trauma exposure (*n*=166; 36.5%) vs. Indirect trauma exposure (*n*=267; 58.7%)^d^ | | | | | | |
| DSM-5 | PCL-5 (*DSM-5*)  -Self-report | A; MLR | χ^2^, RMSEA, CFI, TLI, BIC (A only) | ∆χ^2^, ∆CFI | -No Invariance | - |
| EB | PCL-5 (*DSM-5*)  -Self-report | A; MLR | χ^2^, RMSEA, CFI, TLI, BIC (A only) | ∆χ^2^, ∆CFI | -No Invariance | - |
| Anh | PCL-5 (*DSM-5*)  -Self-report | A-C; MLR | χ^2^, RMSEA, CFI, TLI, BIC (A only) | ∆χ^2^, ∆CFI | A-C | - |
| Hybrid | PCL-5 (*DSM-5*)  -Self-report | A-C; MLR | χ^2^, RMSEA, CFI, TLI, BIC  (A only) | ∆χ^2^, ∆CFI | A-C | - |
| Hoyt et al. (2010); CS; Hispanic (*n*=226; 44.8%) vs. White (*n*=278; 55.2%)^c^ | | | | | | |
| EN | PCL-C (*DSM-IV*)  -Self-report | A-D; ML | χ^2^, RMSEA, CFI, AIC, ECVI | ∆χ^2^, ∆RMSEA, ∆CFI, ∆AIC, ∆ECVI | A, B, D | - |
| Dysphoria | PCL-C (*DSM-IV*)  -Self-report | A-C, D; ML | χ^2^, RMSEA, CFI, AIC, ECVI | ∆χ^2^, ∆RMSEA, ∆CFI, ∆AIC, ∆ECVI | A, B | - |
| 4-Factor EN Model Smith et al.* | PCL-C (*DSM-IV*)  -Self-report | A-D; ML | χ^2^, RMSEA, CFI, AIC, ECVI | ∆χ^2^, ∆RMSEA, ∆CFI, ∆AIC, ∆ECVI | A, B | - |
| Keane et al. (2014); Longitudinal; Baseline (*n*=507; 100%) vs. 8-week post-intervention (*n*=267; 52.7%) vs. 3-month follow-up (*n*=256; 44.9%)^l^ | | | | | | |
| DSM-5 | PCL-5 (*DSM-5)*  -Self-report | A-C; MLR | χ^2^, RMSEA, CFI, TLI, SRMR, BIC | ∆S-B χ^2^ | A, B | - |
| Krause et al. (2007); CS; IPV sample (*n*=396; 49.4%) vs. IPV-services (*n*=405; 50.6%)^i^ | | | | | | |
| Dysphoria | PCL (*DSM-IV*)  -Self-report | A, B, E (Phi); MLR | Y–Bχ^2^, R-RMSEA, R-CFI, R-AIC | No ∆ values | A, B, E (Phi) | - |
| Krause et al. (2007); Longitudinal; IPV-services subsample; T1 (approximately 1 month post-IPV; *n*=405; 100%) vs. T2 (approximately 3 months post-IPV; *n*=326; 80.5%)^e^ | | | | | | |
| Dysphoria | PCL (*DSM-IV*)  -Self-report | A, B, E (Phi); MLR | χ^2^, S-Bχ^2^  (for A only), Y-B χ^2^, R-RMSEA, R-CFI, R-AIC | No ∆ values | A, B, E (Phi) | - |
| Mansfield et al. (2010); CS; Non-deployed (*n*=8,794; 56.4%) vs. Deployed (*n*=6,799; 43.6%)^f^ | | | | | | |
| EN | PCL-C (*DSM-IV*)  -Self-report | A-C; MLR | χ^2^, RMSEA, CFI, SRMR, BIC (A only) | ∆χ^2^ | A, B | Scalar invariance tested but not supported (no specific details for partial invariance results reported) |
| Dysphoria | PCL-C (*DSM-IV*)  -Self-report | A-C; MLR | χ^2^, RMSEA, CFI, SRMR, BIC (A only) | ∆χ^2^ | A, B | Scalar invariance tested but not supported (no specific details for partial invariance results reported) |
| Marshall (2004); CS; English: (*n*=299; 71.4%) vs. Spanish (*n*=120; 28.6%)^c^ | | | | | | |
| EN | PCL-C (*DSM-IV*)  -Self-report | A-C, E; ML | χ^2^, RMSEA, CFI, AIC, NNFI | ∆χ^2^ | A, B | Partial scalar invariance by releasing mean-constraint on item I(a); Partial factor covariance invariance by releasing covariance constraint between H and Av dimensions. |
| Meis et al. (2011); CS; Sample 1 (at T3; 15 months post-return; *n*= 340; 14.9%) vs. Sample 2 (at T2; 12 months post-return; *n*= 1,935; 85.1%)^i^ | | | | | | |
| Dysphoria | PCL (*DSM-IV*)  -Self-report | A, B, E; WLSMV^2^ | χ^2^, RMSEA, CFI, TLI, WRMR | ∆χ^2^ | A, E (Phi) | Partial metric invariance by releasing factor loading-constraint on D(c) |
| Meis et al. (2011); Longitudinal (Sample 1); T1 (1 month pre-deployment; *n*=516; 100%) vs. T2 (2-3 months post-deployment; *n*=423; 82.0%) vs. T3 (15 months post-deployment; *n*=340; 65.9%)^h^ | | | | | | |
| Dysphoria | PCL (*DSM-IV*)  -Self-report | A, B, E; WLSMV^2^ | χ^2^, RMSEA, CFI, TLI, WRMR | ∆χ^2^ | A | Partial metric invariance by releasing the factor loading-constraints on D(f), D(g) D(h), D(i), H(d), I(i), and I(k). Partial Phi invariance tested but not supported |
| Meis et al. (2011); Longitudinal (Sample 2); T1 (During deployment; *n*=2,449; 100%) vs. T2 (12 months post-deployment; *n*=943; 38.5%)^h^ | | | | | | |
| Dysphoria | PCL (*DSM-IV*)  -Self-report | A, B, E; WLSMV^2^ | χ^2^, RMSEA, CFI, TLI, WRMR | ∆χ^2^ | A | Partial metric invariance by releasing factor loading constraints on D(a), D(e), D(f), D(g), H(d), I(j), and I(k). Partial Phi invariance tested but not supported |
| Pietrzak et al. (2014); Longitudinal; Police responders; T1 (average 3.30 years post-9/11; *n*=4,035; 100%) vs. T2 (average 5.70 years post-9/11; *n*=3,631; >90.0%) vs. T3 (average 5.30 years post-9/11; *n*=3,361; >90.0%)^e^ | | | | | | |
| DA | PCL-S (*DSM-IV*)  -Self-report | A-D; MLR | S-Bχ^2^, RMSEA, CFI, TLI, AIC, BIC | ∆S-Bχ^2^, ∆CFI | A-D | - |
| Pietrzak et al. (2014); Longitudinal; Non-traditional responders; T1 (average 3.30 years post-9/11; *n*=6,800; 100%) vs. T2 (average 5.70 years post-9/11; *n*=6,120; >90.0%) vs. T3 (average 5.30 years post-9/11; *n*=6,120; >90.0%)^e^ | | | | | | |
| DA | PCL-S (*DSM-IV*)  -Self-report | A-D; MLR | S-Bχ^2^, RMSEA, CFI, TLI, AIC, BIC | ∆S-Bχ^2^, ∆CFI | A-D | - |
| Simms et al. (2002); CS; Deployed sample 1 (*n=* 948; 27.5%) vs. Deployed sample 2 (*n=*948; 25.7%)^i^ | | | | | | |
| Dysphoria | PCL-M (*DSM-IV*)  -Self-report | A, B; ML | χ^2^, RMSEA, CFI, SRMR, NFI, AGFI | No ∆ values | A, B | - |
| Simms et al. (2002); CS; Deployment history (*n*=1,896; 51.3%) vs. No deployment history (*n*=1,799; 48.7%)^f^ | | | | | | |
| Dysphoria | PCL-M (*DSM-IV*)  -Self-report | A, B; ML | χ^2^, RMSEA, CFI, SRMR, NFI, AGFI | No ∆ values | A, B | - |
| Wang et al. (2012); Longitudinal; T1 (5 months post-earthquake; *n*=403; 100%) vs. T2 (11 months post-earthquake; *n*=403; 100%)^e^ | | | | | | |
| 3-Factor *DSM-IV* | PCL-C (*DSM-IV*)  -Self-report | A-E; MLM | S-Bχ^2^, RMSEA, CFI, TLI, SRMR, BIC | ∆S-Bχ^2^, ∆CFI | A, B, E | - |
| EN | PCL-C (*DSM-IV*)  -Self-report | A-E; MLM | S-Bχ^2^, RMSEA, CFI, TLI, SRMR, BIC | ∆S-Bχ^2^, ∆CFI | A, B, E | - |
| Dysphoria | PCL-C (*DSM-IV*)  -Self-report | A-E; MLM | S-Bχ^2^, RMSEA, CFI, TLI, SRMR, BIC | ∆S-Bχ^2^, ∆CFI | A, B, E | - |
| DA | PCL-C (*DSM-IV*)  -Self-report | A-E; MLM | S-Bχ^2^, RMSEA, CFI, TLI, SRMR, BIC | ∆S-Bχ^2^, ∆CFI | A, B, E | - |
| Wang et al. (2013); CS; Boys (*n*=297; 52.0%) vs. Girls (*n*=274; 48.0%)^a^ | | | | | | |
| Dysphoria | PCL-C (*DSM-IV*)  -Self-report | A-F; MLM | S-Bχ^2^, RMSEA, CFI, TLI, SRMR, BIC | ∆S-Bχ^2^ | A, B, D, E | - |
| EN | PCL-C (*DSM-IV*)  -Self-report | A-F; MLM | S-Bχ^2^, RMSEA, CFI, TLI, SRMR, BIC | ∆S-Bχ^2^ | A, B, D, E | - |
| DA | PCL-C (*DSM-IV*)  -Self-report | A-F; MLM | S-Bχ^2^, RMSEA, CFI, TLI, SRMR, BIC | ∆S-Bχ^2^ | A, B, D, E | - |
| Wang et al. (2017); Longitudinal; Wave 1 (3 months post-blast; *n*=836; 100%) vs. Wave 2 (8 months post-blast; *n*=762; 91.1%)^e^ | | | | | | |
| Hybrid | PCL-5 (*DSM-5*)  -Self-report | A-E; WLSMV^2^ | χ^2^, RMSEA, CFI, TLI | ∆χ^2^, ∆CFI | A-E | - |
| **PCL and PTSD Symptom Scale (PSS)** | | | | | | |
| Elhai et al. (2011); CS; PCL-S (*n*=182; 47.3%) vs. PSS (*n*=203; 52.7%)^g^ | | | | | | |
| EN | PCL-S, PSS  (*DSM-IV*)  -Self-report | A-F; MLM | ∆S-Bχ^2^ | ∆S-Bχ^2^ | B, E, F | - |
| Dysphoria | PCL-S, PSS  (*DSM-IV*)  -Self-report | A-F; MLM | S-Bχ^2^ | ∆S-Bχ^2^ | A, B, F | - |
| **UCLA PTSD Reaction Index-Revised (PTSD-RI)** | | | | | | |
| Armour et al. (2011a); CS; Criterion A2 group (*n*=1,037; 76.1%) vs. Non-Criterion A2 group (*n*=325; 23.9%)^j^ | | | | | | |
| EN | PTSD-RI (*DSM-IV*)  -Self-report | A-F; ML | χ^2^, RMSEA, CFI, TLI, SRMR, BIC (A only) | ∆χ^2^ | A | - |
| Armour et al. (2011b); CS; Boys (*n*=471; 31.8%) vs. Girls (*n*=1,009; 68.2%)^a^ | | | | | | |
| EN | PTSD-RI (*DSM-IV*)  -Self-report | A-F; ML | χ^2^, RMSEA, CFI, TLI, SRMR, BIC (A only) | ∆χ^2^ | A | - |
| Bennett et al. (2014); CS; Boys (*n*=990; 72.6%) vs. Girls (*n*=373; 27.4%)^a^ | | | | | | |
| DA | PTSD-RI (*DSM-IV*)  -Self-report | A, B; ML | χ^2^, RMSEA, CFI, SRMR  (B only) | ∆χ^2^ | A | - |
| Contractor et al. (2013); CS; Pre-adolescents (*n*=3,443; 52.2%) vs. Adolescents (*n*=3,148; 47.8%)^b^ | | | | | | |
| DA | PTSD-RI (*DSM-IV*)  -Self-report | A-F; ML^1^ | χ^2^, CFI, BIC | ∆χ^2^, ∆CFI | A, B, E, F  (based on ∆CFI) | - |
| Contractor et al. (2013); CS; Males *(n*=2,934; 44.5%) vs. Females (*n*=3,657; 55.5%)^a^ | | | | | | |
| DA | PTSD-RI (*DSM-IV*)  -Self-report | A-F; ML^1^ | χ^2^, CFI, BIC | ∆χ^2^, ∆CFI | A-F (based on ∆CFI) | - |
| Contractor et al. (2015); CS; Hispanics (*n*=2,395; 38.3%) vs. Non-Hispanics (*n*=3,853; 61.7%)^c^ | | | | | | |
| DA | PTSD-RI (*DSM-IV*)  -Self-report | A-F; ML^1^ | χ^2^, RMSEA, CFI, TLI, SRMR, BIC | ∆χ^2^, ∆CFI | A-F (based on ∆CFI) | - |
| Contractor et al. (2015); CS; Caucasians (*n*=3,767; 57.2%) vs. Non-Caucasians (*n*=2,824; 42.9%)^c^ | | | | | | |
| DA | PTSD-RI (*DSM-IV*)  -Self-report | A-F; ML^1^ | χ^2^, RMSEA, CFI, TLI, SRMR, BIC | ∆χ^2^, ∆CFI | A-F (based on ∆CFI) | - |
| Nygaard et al. (2012); Longitudinal; T1 (10-11 months post–tsunami; *n*= 133; 100%) vs. T2 (2.5 years post–tsunami; *n*=104; 78.2%)^e^ | | | | | | |
| EN | PTSD-RI (*DSM-IV*)  -Interview | A, B, E (Phi); MLR | χ^2^, RMSEA, CFI, TLI, AIC, ML Discrepancy | ∆χ^2^ | A | - |
| **Impact of Event Scale–Revised (IES-R)** | | | | | | |
| Gargurevich et al. (2009); CS; Fire survivors (*n*=174; 23.5%) vs. University students (*n*=562; 76.4%)^i^ | | | | | | |
| 4-Factor *DSM-IV* with Sleep* | IES-R (*DSM-IV*)  -Interview (survivors)  -Self-report (students) | A, B, E; ML | χ^2^, RMSEA, CFI, AIC | ∆χ^2^ | A, B | Partial factor correlation invariance by releasing the factor correlation-constraint between I and Av |
| King et al. (2009); CS; US students (*n*=235; 43.4%) vs. Israeli ER patients at T1 (average 9.90 days post-ER admission; *n*=306; 56.6%)^c^ | | | | | | |
| 4-Factor *DSM-IV* with Sleep* | IES-R (*DSM-IV*)  -Self-report | A; MLR | χ^2^, RMSEA, CFI, TLI, SRMR, BIC | No ∆ values | A | - |
| 5-Factor EN with Sleep* | IES-R (*DSM-IV*)  -Self-report | A; MLR | χ^2^, RMSEA, CFI, TLI, SRMR, BIC | No ∆ values | A | - |
| 5-Factor Dysphoria with Sleep* | IES-R (*DSM-IV*)  -Self-report | A; MLR | χ^2^, RMSEA, CFI, TLI, SRMR, BIC | No ∆ values | A | - |
| King et al. (2009); Longitudinal; Israel patients; T1 (average 9.90 days post-ER admission; *n*=235; 100%) vs. T2 (average 39.80 days post-ER admission; *n*=235; 100%) vs. T3 (average 168.40 days post-ER admission; *n*=235; 100%)^e^ | | | | | | |
| 4-Factor *DSM-IV* with Sleep* | IES-R (*DSM-IV*)  -Self-report | A, B; MLR | χ^2^, RMSEA, CFI, TLI, SRMR_b/w_, SRMR_w/in_, BIC | ∆χ^2,^ ∆BIC | A | Partial metric invariance by releasing factor-loading constraints on I |
| Suvak et al. (2008); Longitudinal; Wave 2 (approximately 2 months post-9/11; *n*= 685; 100%) vs. Wave 3 (approximately 6 months post-9/11; *n*= 685; 100%)^e^ | | | | | | |
| EN* | IES-R (*DSM-IV-TR*)  -Self-report | A, B, E; ML^1^ | χ^2^, BIC | ∆χ^2^, ∆BIC | A, B | - |
| **Harvard Trauma Questionnaire (HTQ)** | | | | | | |
| Rasmussen et al. (2015); CS; West Africans (*n=*306; 34.9%) vs. Himalayans (*n=*188; 21.4%) vs. Others (*n=*383; 43.6%)^c^ | | | | | | |
| EN | HTQ (*DSM-IV*)  -Self-report | A-C; MLM | LR-χ^2^, RMSEA, TLI, SRMR | ∆LR-χ^2^ | A | - |
| Tay et al. (2017); CS; Males (*n=*1,222; 28.7%) vs. Females (*n=* 3,038; 71.3%)^a^ | | | | | | |
| *DSM-5** | HTQ (*DSM-IV*)  -Interview | A, B, C; WLSMV | χ^2^, RMSEA, CFI, TLI | No ∆ values | A, B, C | - |
| Tay et al. (2017); CS; Sinhalese (*n=*1,326l; 31.1%) vs. Tamil (*n=* 1,952; 45.8%) vs. Moor (*n=* 982; 23.1%)^c^ | | | | | | |
| *DSM-5** | HTQ (*DSM-IV*)  -Interview | A, B, C; WLSMV | χ^2^, RMSEA, CFI, TLI | No ∆ values | A, B, C | - |
| Wind et al. (2017); CS; Indo-Iranian language (*n*=262; 20.9%) vs. Niger-Congo language (*n*=134; 10.7%) vs. Semitic language (*n*=288; 22.9%) vs. South Slavic language (*n*=199; 15.8%) vs. Germanic language (*n*=373; 29.7%)^c^ | | | | | | |
| 3-Factor *DSM-IV* Model (I, hypervigilance, Av)* | HTQ (*DSM-IV*)  -Self-report | A, C, D; WLSMV^4^ | χ^2^, RMSEA, CFI, TLI | ∆χ^2^, ∆CFI | A | Partial scalar invariance established. Partial strict invariance tested but not supported (no information on what parameters were constrained) |
| **Posttraumatic Stress Diagnostic Scale (PDS)** | | | | | | |
| Baschnagel et al. (2005); Longitudinal; T1 (1 month post-9/11 attack; *n*=528; 100%) vs. T2 (3 months post-9/11 attack; *n*=528; 100%)^e^ | | | | | | |
| Dysphoria | PDS (*DSM-IV*)  -Self-report | A, B; MLM^4^ | χ^2^, RMSEA, CFI, TLI (A only) | ∆χ^2^ | A | - |
| Ullman et al. (2008); CS; Black (*n=*418; 43.2%) vs. White (*n*=377; 39.0%)^c^ | | | | | | |
| EN | PDS *(DSM-IV*)  -Self-report | A, B; WLSMV | χ^2^, RMSEA, CFI, TLI, SRMR | ∆χ^2^ | -No invariance | - |
| Ullman et al. (2008); CS; High school or less (*n=*268; 27.7%) vs. Some college and beyond (*n=*699; 72.3%)^k^ | | | | | | |
| EN | PDS (*DSM-IV*)  -Self-report | A, B; WLSMV | χ^2^, RMSEA, CFI, TLI, SRMR | ∆χ^2^ | -No invariance | - |
| **Child PTSD Symptom Scale (CPSS)** | | | | | | |
| Hukkelberg (2014); CS; Boys (*n*=152; 39.0%) vs. Girls (*n*=238; 61.0%)^a^ | | | | | | |
| Dysphoria | CPSS (*DSM-IV*)  -Self-report | A-C, E, F; MLM^2^ | S-Bχ^2^, CFI, TLI, RMSEA | ∆S-Bχ^2^ | A, B, E | - |
| EN | CPSS (*DSM-IV*)  -Self-report | A-C, E, F; MLM^2^ | S-Bχ^2^, CFI, TLI, RMSEA | ∆S-Bχ^2^ | A-C, E | - |
| **PTSD Symptom Scale (PSS)** | | | | | | |
| Lommen et al. (2014); Longitudinal; Dutch sample 1 – Afghanistan deployment; T1 (2 months pre-deployment; *n*=249; 100%) vs. T2 (2 months post-deployment; *n*=241; 96.8%)^h^ | | | | | | |
| 1-Factor | PSS (*DSM-IV*)  -Self-report | A-C, Model  with only threshold invariance; WLSMV & MLR (for AIC & BIC)^3^ | χ^2^, RMSEA, CFI, TLI, AIC, BIC | ∆χ^2^ | A, B | Partial threshold invariance supported in (l), (m), and (o) (constrained one item threshold at a time) |
| Lommen et al. (2014); Longitudinal; Dutch sample 2 – Iraq deployment; T1 (pre-deployment; *n*=291; 95.4%) vs. T2 (5 months post-deployment; *n*=242; 79.3%)^h^ | | | | | | |
| 1-Factor | PSS (*DSM-IV*)  -Self-report | A-C, Model examining  only threshold invariance; WLSMV & MLR (for AIC & BIC)^3^ | χ^2^, RMSEA, CFI, TLI, AIC, BIC | ∆χ^2^ | A, B | Partial threshold invariance supported in (l), (m), and (o) (constrained one item threshold at a time) |
| Lommen et al. (2014); CS; Deployment history; With (*n=*268; 48.3%) vs. Without (*n=*286; 51.6%)^f^ | | | | | | |
| 1-Factor | PSS (*DSM-IV*)  -Self-report | A-C  (only thresholds); WLSMV & MLR (for AIC & BIC)^3^ | AIC, BIC | ∆χ^2^ | A, B | - |
| **Davidson Trauma Scale (DTS)** | | | | | | |
| Mason et al. (2013); Longitudinal; Among burn patients, 1 month post-baseline assessment (*n*=299; 100%) vs. 6 month post-baseline assessment (*n*=242; 80.9%) vs. 12 month post-baseline assessment (*n*=212; 70.9%) vs. 24 month post-baseline assessment (*n*=171; 57.2%)^e^ | | | | | | |
| Dysphoria | DTS (*DSM-IV*)  -Self-report | A, B; MLR | χ^2^, RMSEA, CFI, TLI, SRMR_b/w_, SRMR_w/in_, BIC | ∆χ^2^, ∆BIC | A, B | - |
| EN | DTS (*DSM-IV*)  -Self-report | A, B; MLR | χ^2^, RMSEA, CFI, TLI, SRMR_b/w_, SRMR_w/in_, BIC | ∆χ^2^, ∆BIC | A | Partial metric invariance was supported by releasing constraints on I and Av factor items. |
| McDonald et al. (2008); CS; OEF/OIF veteran research participants (*n=*313; 21.7%) vs. Post-Vietnam veterans (*n=*313; 21.7%)^i^ | | | | | | |
| EN | DTS (*DSM-IV*)  -Self-report | A, B;  WLSMV | S-Bχ^2^, RMSEA, CFI, SRMR, AIC | ∆S-Bχ^2^ | A | - |
| McDonald et al. (2008); CS; OEF/OIF veteran research participants (*n=*313; 21.7%) vs. Vietnam veterans (*n=*814; 56.5%)^i^ | | | | | | |
| EN | DTS (*DSM-IV*)  -Self-report | A, B;  WLSMV | S-Bχ^2^, RMSEA, CFI, SRMR, AIC | ∆S-Bχ^2^ | A | - |
| McDonald et al. (2008); CS; Post-Vietnam veterans (*n=*313; 21.7%) vs. Vietnam veterans (*n=*814; 56.5%)^i^ | | | | | | |
| EN | DTS (*DSM-IV*)  -Self-report | A, B;  WLSMV | S-Bχ^2^, RMSEA, CFI, SRMR, AIC | ∆S-Bχ^2^ | A, B | - |
| **Clinician-Administered PTSD Scale (CAPS)** | | | | | | |
| Contractor et al. (2017); Longitudinal; CAPS; T0 (1 month pre-deployment; *n*=834; 100%) vs. T2 (5 months post-deployment; *n*=310; 37.2%) vs. T3 (8 months post-deployment; *n*=272; 32.6%)^h^ | | | | | | |
| EN (parceled) | CAPS (*DSM-IV*)  -Interview | A-C; MLR | χ^2^, RMSEA, CFI, TLI, SRMR, BIC | ∆χ^2^, ∆CFI | A, B | Partial scalar invariance by releasing the intercept and factor-loading constraints on I(a) and N(c) |
| 1-Factor | CAPS (*DSM-IV*)  -Interview | A-C; MLR | χ^2^, RMSEA, CFI, TLI, SRMR, BIC | ∆χ^2^, ∆CFI | A, B | Partial scalar invariance by releasing factor loading and intercept constraints on Av and N |
| Elhai et al. (2010); CS; PTSD frequency format (*n*=747; 100%) vs. PTSD intensity format (*n*=747; 100%)^g^ | | | | | | |
| EN | CAPS (*DSM-IV*)  -Interview (phone) | A-D; MLM | S-Bχ^2^, BIC | ∆S-Bχ^2^, ∆BIC | B | - |
| **Posttraumatic Stress Disorder Questionnaire (PTSD-Q)** | | | | | | |
| Hetzel-Riggin (2009); CS; Child sexual abuse (*n*=254; 10.7%) vs. Child physical abuse (*n*=406; 17.1%) vs. Adult sexual assault (*n*=577; 24.3%) vs. Adult physical assault (*n*=299; 12.6%) vs. Multiple abuse (*n*=842; 35.4%)^d^ | | | | | | |
| Dysphoria | PTSD-Q (*DSM-IV*)  -Self-report | A, B, D, E; MLR | χ^2^, S-Bχ^2^, Y-Bχ^2^, R-RMSEA, R-CFI, R-CAIC, R-NNF | No ∆ values | A, B, D, E | - |
| **Psychological Reactions Following International Missions Questionnaire (PRIM)** | | | | | | |
| Karstoft et al. (2017); CS; Cohort 1 (returning from deployment in 2009; *n*=334; 54.6%) vs. Cohort 2 (returning from deployment in 2013; *n*=278; 45.4%)^i^ | | | | | | |
| 1-Factor | PRIM (*DSM-IV*)  -Self-report | A-C;  WLSMV^4^ | χ^2^, RMSEA, CFI, TLI | ∆χ^2^ | A-C | - |
| **Diagnostic Interview Schedule – PTSD Module** | | | | | | |
| Saul et al., 2008; CS; Males (*n*=824; 52.1%) vs. Females (*n*=757; 47.9%)^a^ | | | | | | |
| EN | -Diagnostic Interview Schedule – PTSD Module (modified; *DSM-IV*)  -Interview | A, B; NR | χ^2^, RMSEA, CFI | ∆χ^2^ | A | - |
| Saul et al., 2008; CS; Children ages 12-14 (*n*=NR) vs. Children ages 15-17 (*n*=NR)^b^ | | | | | | |
| EN | -Diagnostic Interview Schedule – PTSD Module  (modified; *DSM-IV*)  -Interview | A, B; NR | χ^2^, RMSEA, CFI | ∆χ^2^ | A | - |
| Saul et al., 2008; CS; Violent traumatic stressors (*n*=NR) vs. Non-violent traumatic stressors (*n*=NR)^d^ | | | | | | |
| EN | -Diagnostic Interview Schedule – PTSD Module  (modified; *DSM-IV*)  -Interview | A, B; NR | χ^2^, RMSEA, CFI | ∆χ^2^ | A | - |
| **National Survey of Adolescents – PTSD Module** | | | | | | |
| Sumner et al. (2014); CS; Males (*n*=980; 49.0%) vs, Females (*n*=1,019; 51.0%)^a^ | | | | | | |
| DA | -National Survey of Adolescents – PTSD Module  (*DSM-IV*)  -Interview (phone) | A-F; WLSMV^4^ | χ^2^, RMSEA, CFI, TLI | ∆χ^2^ | A-E | - |
| Sumner et al. (2014); CS; Ages 12-14 (*n*=975; 48.9%) vs. Ages 15-17: (*n*=1,020; 51.1%)^b^ | | | | | | |
| DA | -National Survey of Adolescents – PTSD Module  (*DSM-IV*)  -Interview (phone) | A-F; WLSMV^4^ | χ^2^, RMSEA, CFI, TLI | ∆χ^2^ | A-E | - |
| **Mini-International Neuropsychiatric Interview 6 (MINI-6) – PTSD Module** | | | | | | |
| Zelazny et al. (2015); CS; Criterion A trauma (*n*=310; 52.2%) vs. Non-criterion A trauma (*n*=284; 47.8%)^d^ | | | | | | |
| Dysphoria* | -MINI-6 – PTSD Module (modified; *DSM-5*)  -Interview | A, C; WLSMV & MLR (for AIC & BIC)^3^ | RMSEA, CFI, TLI | - | A, C (assumed metric invariance) | - |
| Hybrid | -MINI-6 – PTSD Module (modified; *DSM-5*)  -Interview | A, C; WLSMV & MLR (for AIC & BIC)^3^ | RMSEA, CFI, TLI |  | A, C (assumed metric invariance) | - |

*Note*. CS is cross-sectional; NR is not reported; PCL-5 is the PTSD Checklist for DSM-5; PCL-C is the PTSD Checklist-Civilian; PCL-M is the PTSD Checklist-Military; PCL-S is the PTSD Checklist-Specific Stressor; IPV is interpersonal violence; OEF/OIF is Operation Enduring Freedom/Operation Iraqi Freedom; ER is emergency room; * signifies a modified model; Models: A is configural/form invariance; B is metric/weak factorial invariance; C is scalar/strong factorial invariance; D is strict factorial invariance; E is equality of factor variances and covariances (phi invariance); F is factor means invariance; EN is the Emotional Numbing Model; DA is Dysphoric Arousal Model; EB is Externalizing Behaviors Model; Anh is Anhedonia Model.

∆ is change in; RMSEA is root mean square error of approximation; CFI is comparative fit index; TLI is Tucker-Lewis index; SRMR is standardized root mean square residual; BIC is Bayesian information criterion; AIC is Akaike information criterion; SRMR_w/in_ is SRMR within; SRMR_b/w_ is SRMR between; ECVI is expected cross-validation index; AGFI is adjusted goodness of fit index; WRMR is weighted root-mean-square residual; R-RMSEA is robust RMSEA; R-CFI and CFI-R is robust CFI ; R-AIC is robust AIC; χ^2^ is chi-square; CAIC is the consistent version of AIC; R-CAIC is the robust consistent AIC; NFI is Bentler-Bonett normed fit index; NNF/NNFI is non-normed fit index; R-NNF is the robust NFI; LR is corrected likelihood ratio; S-B is Satorra-Bentler; Y-B is Yuan-Bentler; ML is maximum likelihood; MLR is ML estimation with robust standard errors; MLM is ML estimation with mean adjustment; WLSMV is mean- and variance- adjusted weighted least squares estimations.

Superscript numbers signify how data was treated: ^1^ is continuous; ^2^ is ordinal; ^3^ is dichotomous; ^4^ is categorical (if no superscript, authors did not report how data was treated); superscript letters signify how subgroups comparisons were classified: ^a^ is gender; ^b^ is age; ^c^ is cultural/lingual; ^d^ is types/levels of TE; ^e^ is time-points post-TE; ^f^ is deployed vs. not deployed; ^g^ is administration mode/type of measures; ^h^ is pre-/during and post-deployment; ^i^ is diverse samples; ^j^ is psychopathology/diagnosis; ^k^ is education; ^l^ is intervention- time-points.

I is intrusion/re-experiencing; Av is avoidance; N is numbing; D is dysphoria; H is hyperarousal/arousal; I/Av is intrusion/avoidance; N/H is numbing/hyperarousal; (a) is physiological reactivity to trauma cues; (b) is feeling numb; (c) is foreshortened future; (d) is hypervigilance; (e) is traumatic amnesia; (f) is anhedonia; (g) is social detachment; (h) is restricted range of affect; (i) is anger/irritability; (j) is intrusive memories; (k) is psychological reactivity to trauma cue; (l) is acting/feeling as if trauma was recurring; (m) is sleep difficulties; (n) is concentration difficulties.

Supplemental Table 5. *Count of obtained vs. examined invariance (number of MG analyses) for PTSD models, measures, and subgroup comparisons*

|  | **A** | **B** | **Partial B** | **C** | **Partial C** | **D** | **Partial D** | **E** | **Partial E** | **F** |
| --- | --- | --- | --- | --- | --- | --- | --- | --- | --- | --- |
| **PTSD factor-analytical models (*count of obtained/examined invariance; %)*** | | | | | | | | | | |
| *DSM-IV* 1-factor | 6/6* | 6/6* | 0/0 | 1/6 (16.7) | 4/4* | 0/0 | 0/0 | 0/0 | 0/0 | 0/0 |
| *DSM-IV* 3- factor# | 6/6* | 2/4 (50) | 1/1* | 0/2 (0) | 1/1* | 0/2 (0) | 0/1 (0) | 1/3 (33.3) | 1/1* | 0/0 |
| *DSM-IV* EN# | 29/33 (87.9) | 16/32 (50) | 3/3* | 3/19 (15.8) | 3/4 (75) | 3/13 (23.1) | 0/0 | 4/15 (26.7) | 1/1* | 2/10 (20) |
| *DSM-IV* Dys# | 25/25* | 18/24 (75) | 3/3* | 3/13 (23.1) | 1/2 (50) | 3/10 (30) | 0/0 | 7/16 (43.8) | 0/0 | 2/8 (25) |
| *DSM-IV* DA | 11/11* | 10/11 (90.9) | 0/0 | 7/10 (70) | 0/0 | 8/10 (80) | 0/0 | 8/8* | 0/0 | 4/7 (57.1) |
| *DSM-5* 4-factor# | 3/5 (60) | 3/3* | 0/0 | 2/3 (66.7) | 0/0 | 0/0 | 0/0 | 0/0 | 0/0 | 0/0 |
| *DSM-5* Hybrid | 5/5* | 5/5* | 0/0 | 5/5* | 0/0 | 2/2* | 0/0 | 2/2* | 0/0 | 0/1 (0) |
| *DSM-5* EB | 0/2 (0) | 0/0 | 0/0 | 0/0 | 0/0 | 0/0 | 0/0 | 0/0 | 0/0 | 0/0 |
| *DSM-5* Anh | 2/2* | 2/2* | 0/0 | 2/2* | 0/0 | 0/0 | 0/0 | 0/0 | 0/0 | 0/0 |
| Taylor et al. | 1/1* | 0/1 (0) | 1/1* | 0/0 | 0/0 | 0/0 | 0/0 | 0/0 | 0/0 | 0/0 |
| **Self-report measures (*count of obtained/examined invariance; %)*** | | | | | | | | | | |
| PCL | 47/51 (92.2) | 36/47 (76.6) | 6/6* | 12/35 (34.3) | 4/6 (66.7) | 10/24 (41.7) | 0/0 | 12/28 (42.9) | 1/1* | 2/14 (14.3) |
| PTSD-RI | 8/8* | 4/8 (50) | 0/0 | 3/6 (50) | 0/0 | 3/6 (50) | 0/0 | 4/7 (57.1) | 0/0 | 4/6 (66.7) |
| IES-R | 6/6* | 2/3 (66.7) | 1/1* | 0/0 | 0/0 | 0/0 | 0/0 | 0/2 (0) | 1/1* | 0/0 |
| HTQ | 4/4* | 2/3 (66.7) | 0/0 | 2/4 (50) | 1/1* | 0/1 (0) | 0/1 (0) | 0/0 | 0/0 | 0/0 |
| PDS | 1/3 (33.3) | 0/3 (0) | 0/0 | 0/0 | 0/0 | 0/0 | 0/0 | 0/0 | 0/0 | 0/0 |
| CPSS | 2/2* | 2/2* | 0/0 | 1/2 (50) | 0/0 | 0/0 | 0/0 | 2/2* | 0/0 | 0/2 (0) |
| PSS-SR | 3/3* | 3/3* | 0/0 | 0/3 (0) | 2/2* | 0/0 | 0/0 | 0/0 | 0/0 | 0/0 |
| DTS | 5/5* | 2/5 (40) | 1/1* | 0/0 | 0/0 | 0/0 | 0/0 | 0/0 | 0/0 | 0/0 |
| PTSD-Q | 1/1* | 1/1* | 0/0 | 0/0 | 0/0 | 1/1* | 0/0 | 1/1* | 0/0 | 0/0 |
| PRIM | 1/1* | 1/1* | 0/0 | 1/1* | 0/0 | 0/0 | 0/0 | 0/0 | 0/0 | 0/0 |
| **Clinician-administered measures (*count of obtained/examined invariance; %)*** | | | | | | | | | | |
| CAPS | 2/3 (66.7) | 3/3* | 0/0 | 0/3 (0) | 2/2* | 0/1 (0) | 0/0 | 0/0 | 0/0 | 0/0 |
| Diagnostic Interview Schedule – PTSD Module | 3/3* | 0/3 (0) | 0/0 | 0/0 | 0/0 | 0/0 | 0/0 | 0/0 | 0/0 | 0/0 |
| National Survey of Adolescents – PTSD Module | 2/2* | 2/2* | 0/0 | 2/2* | 0/0 | 2/2* | 0/0 | 2/2* | 0/0 | 0/2 (0) |
| MINI-6 – PTSD Module | 2/2* | 2/2* | 0/0 | 2/2* | 0/0 | 0/0 | 0/0 | 0/0 | 0/0 | 0/0 |
| **Subgroup comparisons (*count of obtained/examined invariance; %)*** | | | | | | | | | | |
| Gender | 14/16 (87.5) | 11/14 (78.6) | 0/0 | 7/12 (58.3) | 0/0 | 6/7 (85.7) | 0/0 | 8/9 (88.9) | 0/0 | 1/9 (11.1) |
| Age | 3/3* | 2/3 (66.7) | 0/0 | 1/2 (50) | 0/0 | 1/2 (50) | 0/0 | 2/2* | 0/0 | 1/1* |
| Cultural/lingual | 12/13 (92.3) | 7/9 (77.8) | 0/0 | 3/9 (33.3) | 2/2* | 3/6 (50) | 0/1 (0) | 2/3 (66.7) | 1/1* | 2/2* |
| Types/levels of TE | 12/14 (85.7) | 9/12 (75) | 0/0 | 6/10 (60) | 0/0 | 3/7 (42.9) | 0/0 | 1/7 (14.3) | 0/0 | 2/6 (33.3) |
| Time-points post-TE | 17/17* | 12/17 (70.6) | 2/2* | 3/7 (42.9) | 0/0 | 3/7 (42.9) | 0/0 | 6/11 (54.5) | 0/0 | 0/0 |
| Deployed vs. not deployed | 6/6* | 4/6 (66.7) | 0/0 | 2/5 (40) | 0/2 (0) | 0/2 (0) | 0/0 | 0/2 (0) | 0/0 | 0/2 (0) |
| Administration mode/type of measures | 2/4 (50) | 4/4* | 0/0 | 0/4 (0) | 1/1* | 0/3 (0) | 0/0 | 1/2 (50) | 0/0 | 2/2* |
| Pre-/during and post-deployment | 8/8* | 5/8 (62.5) | 3/3* | 0/6 (0) | 6/6* | 0/0 | 0/0 | 0/2 (0) | 0/0 | 0/0 |
| Diverse samples | 8/8* | 5/8 (62.5) | 1/1* | 1/1* | 0/0 | 0/0 | 0/0 | 2/3 (66.7) | 1/1* | 0/0 |
| Psychopathology/diagnosis | 5/5* | 2/5 (40) | 2/2* | 0/3 (0) | 0/0 | 0/3 (0) | 0/0 | 0/3 (0) | 0/0 | 0/3 (0) |
| Education | 0/1 (0) | 0/1 (0) | 0/0 | 0/0 | 0/0 | 0/0 | 0/0 | 0/0 | 0/0 | 0/0 |
| Intervention- time-points | 1/1* | 1/1* | 0/0 | 0/1 (0) | 0/0 | 0/0 | 0/0 | 0/0 | 0/0 | 0/0 |

*Note.* PTSD-RI is the UCLA PTSD Reaction Index-Revised; PCL-5 is the PTSD Checklist for DSM-5; PCL is the PTSD Checklist; PCL-C is the PTSD Checklist-Civilian; PCL-M is the PTSD Checklist-Military; PCL-S is the PTSD Checklist-Specific Stressor; CAPS is the Clinician-Administered PTSD Scale; IES-R is the Impact of Event Scale–Revised; PTSD-Q is the Posttraumatic Stress Disorder Questionnaire; DTS is the Davidson Trauma Scale; HTQ is the Harvard Trauma Questionnaire; PDS is the Posttraumatic Stress Diagnostic Scale; PSS is the PTSD Symptom Scale; CPSS is the Child PTSD Symptom Scale; PRIM is the Psychological Reactions Following International Missions Questionnaire, MINI-6 is the Mini-International Neuropsychiatric Interview 6; TE is traumatic event; DA is Dysphoric Arousal Model; EB is Externalizing Behaviors Model; EN is Emotional Numbing Model; Dys is Dysphoria Model; Anh is Anhedonia Model; A - configural/form invariance; B - metric/weak factorial invariance; C - scalar/strong factorial invariance; D - strict factorial invariance; E - equality of factor variances and covariances (phi invariance); F - factor means invariance; # indicates inclusion of modified models; * indicates 100%.
